# Ecological Niche Modeling Predicts Alarming Impacts of Global Climate Change on Economically Important Neotropical Trees

**DOI:** 10.1002/ece3.72105

**Published:** 2025-09-23

**Authors:** Catarina S. Carvalho, Raquel Moura Machado, Maristerra R. Lemes, Domingos Cardoso

**Affiliations:** ^1^ Instituto de Pesquisas Jardim Botânico do Rio de Janeiro Rio de Janeiro Rio de Janeiro Brazil; ^2^ Laboratório de Genética e Biologia Reprodutiva de Plantas (LabGen) Instituto Nacional de Pesquisas da Amazônia, Coordenação de Biodiversidade Manaus Amazonas Brazil; ^3^ Universidade Federal do Maranhão ‐ Centro de Ciências de Grajaú Grajaú Maranhão Brazil; ^4^ Programa de Pós‐Graduação em Biodiversidade e Evolução (PPGBioEvo), Instituto de Biologia Universidade Federal da Bahia Salvador Bahia Brazil

**Keywords:** Amazonia, climate change, conservation, *Dipteryx*, Leguminosae, South America

## Abstract

The neotropical papilionoid legume genus *Dipteryx* comprises tree species widely used for seed harvesting and logging. In Amazonia, all eight known *Dipteryx* species are internationally recognized in the timber trade as *cumaru*, whereas two others, known as *tonka* beans, are appreciated for their aromatic seeds. Non‐Amazonian species also have uses, such as *baru* nuts ( *D. alata* ) from the Cerrado and *fava‐de‐morcego* (*D*. *lacunifera*) from the Caatinga with its edible seeds and dense wood. Amazonia is already highly affected by uncontrolled exploitation and deforestation, which will exacerbate the expected future scenarios of climate change due to severe drought and flooding. These environmental catastrophes have the potential to cause mass species extinction and severely hit vulnerable Amazonian urban populations, Indigenous people, and traditional communities that derive their livelihood from the forest. Here, we assess how the near‐term future (2021–2040) global climate change may affect the distribution of *Dipteryx* species under the moderate and worse greenhouse gas emission scenarios. Additionally, we incorporate insights from the Last Glacial Maximum global changes to enhance our understanding. The ecological niche modeling revealed that while the potential distribution of most Amazonian species remains stable despite global climate change, species with restricted distributions are more vulnerable to global warming. Furthermore, the great ecological predilection of *Dipteryx* species for wet settings makes them reliant on healthy forest ecosystems. The pressures of logging and deforestation pose significant threats to their survival and to Amazonian biodiversity as a whole.

## Graphical abstract

Widespread Amazonian *Dipteryx* species remained stable during both the LGM and future, but logging and deforestation pressures within Amazonian habitats pose significant threats to their survival. *Dipteryx* species depend on healthy forest ecosystems for their survival and conservation, and species with restricted distribution show sensitivity to global warming. Cerrado‐inhabiting *Dipteryx* species expand toward ecotonal areas between the Cerrado and humid forests, and the Caatinga‐inhabiting species is projected to lose up to 40% of its potential distribution.

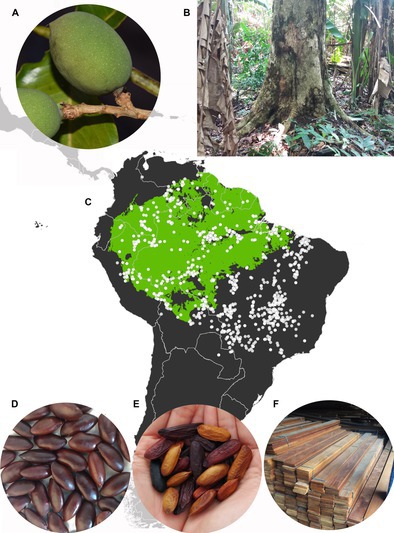

## 1 Introduction

**FIGURE 1:**
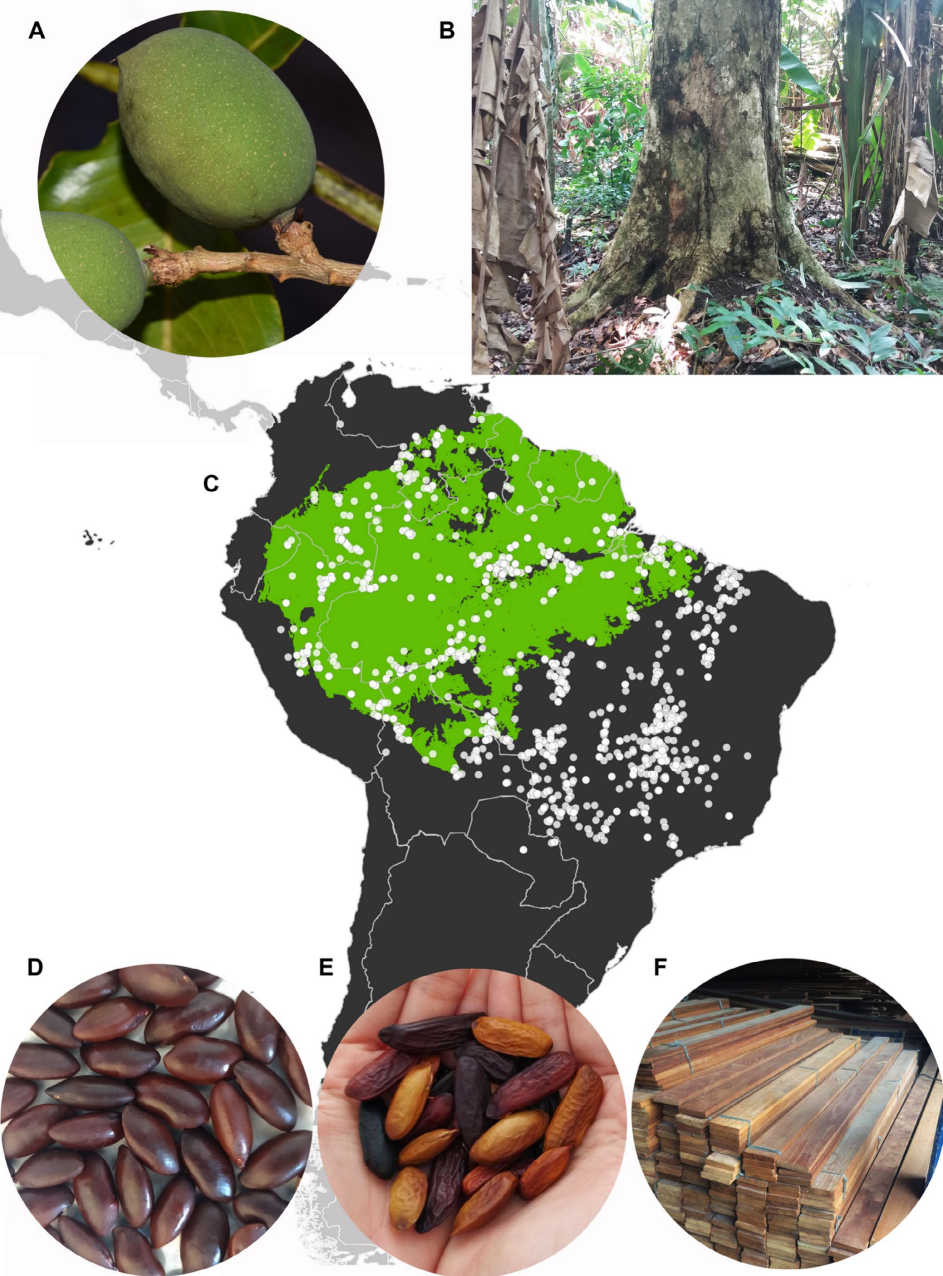
General aspects of *Dipteryx* species. (A) Drupe of *D. odorata* . (B) Habit of *D. odorata* . (C) South American distribution of *Dipteryx* species (see Figure S1 for individual distributions of *D. alata* , *D*. *charapilla*, *D. ferrea* , *D*. *lacunifera*, *D. magnifica* , *D. micrantha* , *D. odorata* , *D*. *polyphylla*, *D. punctata* , and *D. rosea* ). The Amazonian rainforest is delimited in green (sensu Cardoso et al. 2017). (D) Seeds of *D*. *alata*, commercialized under the name of *baru* nuts. (E) Seeds of *D. odorata* and *D. punctata* , commercialized under the name of *tonka* beans. (F) *Cumaru*'s timber that could be from any of the eight Amazonian species of *Dipteryx*. Photos: (A) D. Cardoso; (B) C. Carvalho; (D) A. Ribeiro; (E) T. Cabral; (F) from http://www.bressaninimadeiras.com.br/imagens/produto/grande/fc4ic5fmaa1r_26938167_1603296976415868_1199241512_o.jpg.

*Cumarus* are emergent trees distributed throughout Amazonia and are recognized for their hard, dense wood and generally aromatic seeds. This common name has been applied to eight species of the papilionoid legume genus *Dipteryx* Schreb. (Leguminosae or Fabaceae) and may vary depending on the South American region (e.g., *cumarurana*, *cumaru‐da‐várzea*, *cumaru‐da‐terra firme*, or *shihuahuacu*) (Carvalho, Cardoso, and Lima 2024). The *cumarus* are associated with economic activities such as timber exploitation (all eight species) and the harvesting and sale of seeds, where they are widely used as food flavorings, perfumery, and skincare, and the species are also recognized as *tonka* beans [ *D. odorata* (Aubl.) Forsyth f. and *D. punctata* (S.F.Blake) Amshoff] (Figure 1). However, despite several species being potentially linked to both activities, only the widely distributed *D. odorata* is officially recognized by agencies and companies responsible for regulating exploitation (Putzel 2009; IBAMA 2019; Lush 2024; Natura 2024; Carvalho, Cardoso, and Lima 2024). While the harvesting and sale of *tonka* beans is a small‐scale activity that provides income for Indigenous and traditional Amazonian communities (e.g., *quilombolas* [Afro‐descendant people] and *ribeirinhos* [riverine people]), *cumaru* timber exploitation is a large‐scale activity that directly drives forest habitat loss. Therefore, it is important to develop conservation, impact mitigation, and sustainable use strategies to avoid the risk of species extinction (IBAMA 2019; Brasil 2023). Recently, all internationally traded Amazonian timber species of *Dipteryx* were included in Appendix II of CITES, that is, species not currently threatened but at risk of extinction due to uncontrolled trade (Convention on International Trade in Endangered Species of Wild Fauna and Flora; https://cites.org/eng/app/appendices.php). Non‐Amazonian species of *Dipteryx* also have associated human uses, such as the *baru* (*D. alata* Vogel) found in the fire‐prone savanna vegetation of the Brazilian Cerrado, whose fruits and seeds are highly valued as food; and *D. lacunifera* Ducke, a species from the Caatinga seasonally dry woodlands of northeastern Brazil, with timber potential and seeds used as food, in soap production, and as forage (Ribeiro et al. 2012; Kermath et al. 2014; Flynn 2018; Carvalho, Cardoso, and Lima 2024).

The genus *Dipteryx* (12 species), along with *Monopteryx* Spruce ex Benth. (three species), *Pterodon* Vogel (four species), and *Taralea* Aubl. (four species), comprises the Dipterygeae clade, one of the oldest lineages of the species‐rich Papilionoideae subfamily of legumes (Cardoso et al. 2012, 2015; Carvalho, Lima, et al. 2023; Cai et al. 2025). The greatest diversity of *Dipteryx* species is found in humid forests such as Amazonia, the Caribbean forests of South and Central America, and the Atlantic Forest (Figure 1 and Figure S1; Carvalho, Cardoso, and Lima 2024). However, individuals of the genus *Dipteryx* are also widely distributed in the Cerrado and Caatinga (Figures 1 and Figure S1; Carvalho, Cardoso, and Lima 2024).

Amazonia is considered one of the most ecologically important regions, primarily because it contains the largest area of continuous rainforest, estimated at 5.79 million km^2^, and provides crucial ecosystem services, such as climate regulation through carbon storage and sequestration (ter Steege et al. 2015; Strand et al. 2018). However, the vast Amazonian rainforests are far from homogeneous, exhibiting great variation in rainfall regimes, soils of different geological origins, and vegetation types, including tall‐canopy forests, open low‐vegetation habitats (*campinas* and *campinaranas*), and seasonally flooded forests (*igapós* and *várzeas*) (Hughes et al. 2013; Moraes et al. 2021). Despite its immense biodiversity, the region remains poorly understood due to the high costs of field campaigns, funding demands, and limited research budgets (Oliveira et al. 2016; Magnusson et al. 2016; Hopkins 2019; Guimarães et al. 2024; Stegmann et al. 2024). In Brazil, the country with the largest share of Amazonia, it is estimated that by 2050, between 250,000 and 900,000 km^2^ of under‐sampled areas will be lost to deforestation, leading to the extinction of species before they are even documented (Stropp et al. 2020; Carvalho, Resende, et al. 2023). The unchecked exploitation and deforestation of Amazonia have become even more alarming under the expected future global warming scenarios, as human activities have already driven an increase in the average atmospheric temperature by about 0.9°C [0.84–1.10] (IPCC 2021). Increasing droughts or unusual wet‐dry cycles could trigger mass mortality and impact other moisture‐dependent biomes, such as the La Plata River Basin and Pantanal (Martinez and Dominguez 2014; Marengo et al. 2016; IPCC 2021; Flores et al. 2024). Furthermore, land‐use activities, such as intentional fires, may further exacerbate climate change effects (Leite‐Filho et al. 2019; IPCC 2021; Cano et al. 2022; Flores et al. 2024). Most of the 40 million people living in Amazonia will also face the consequences of severe droughts and floods, particularly vulnerable urban populations, Indigenous peoples, and traditional communities. These groups, whose livelihoods depend directly on Amazonian biodiversity, are disproportionately affected by its loss (Science Panel for the Amazon 2021).

Given the distribution of *Dipteryx* species, we consider them an informative model for understanding how climate change may impact tree distribution across distinct Amazonian habitats and South American biomes. Additionally, assessing their potential distributions—area predicted to be suitable for a species based on its ecological niche and environmental variables—during the Last Glacial Maximum (LGM) can provide valuable insights into species resilience and stability under global climate change, shedding light on their historical and future distribution patterns. These projections are not only essential for guiding conservation strategies, but also for anticipating the socioeconomic impacts of near‐term climate change (next 20 years). Many *Dipteryx* species are resources for the timber industry, which may be affected by habitat shifts and loss of suitable areas. Furthermore, traditional and Indigenous communities that rely on *Dipteryx* species for food, medicine, and income may also face challenges due to environmental changes. Given the existing biogeographic hypotheses for the Dipterygeae clade (Carvalho, Lima, et al. 2024), we also discuss how niche conservatism in *Dipteryx* species may explain the shifts observed in their potential distributions under global climate change from the LGM to the near‐term future.

## 2 Data and Method

### 2.1 Occurrence Data

For this study, we selected *Dipteryx* species distributed across South America. The recently described *D*. *hermetopascoaliana* C.S.Carvalho, H.C.Lima & D.B.O.S.Cardoso, which is endemic to the Atlantic Forest, was excluded due to its limited occurrence records and lack of distribution data (Carvalho et al. 2022; Carvalho, Cardoso, and Lima 2024). As a result, we evaluated 10 species of *Dipteryx*: eight distributed across Amazonia [*D*. *charapilla* (J.F.Macbr.) Ducke, *D. ferrea* (Ducke) Ducke, *D. magnifica* (Ducke) Ducke, *D. micrantha* Harms, *D. odorata* , *D*. *polyphylla* Huber, *D. punctata* , and *D. rosea* Spruce ex Benth.], one in the Cerrado ( *D. alata* ), and one in the Caatinga (*D*. *lacunifera* Ducke) (Figure 1 and Figure S1).

Initially, occurrence data were downloaded from the Global Biodiversity Information Facility (GBIF; https://www.gbif.org/), Jabot (https://jabot.jbrj.gov.br/), and speciesLink (https://specieslink.net/). Additionally, records from herbaria not included in these databases (e.g., HH, IAN, and MG) were manually added. The data were then standardized, duplicates were removed, and coordinates were verified. Species identifications have been curated by the first author continuously since 2016. For this study, we compiled a database containing 1269 records from the following herbaria (abbreviations follow Index Herbariorum; Thiers, continuously updated): ALCB, BR, CEN, COAH, IBGE, HH, HUEFS, IAN, INPA, LISU, K, MG, MO, NY, P, R, RB, RON, TEPB, UB, UFACPZ, and US (Table S1).

### 2.2 Ecological Niche Modeling

#### 2.2.1 Climate Variables

Our approach follows the standards for ecological niche models (ENMs) in biodiversity assessments, as suggested by Araújo et al. (2019). For the present estimations, we downloaded 18 climate variables from WorldClim 2 (Fick and Hijmans 2017), along with monthly averages of maximum, minimum, and mean temperature, and monthly precipitation at a resolution of 30 arc sec (~0.0083°) based on historical climate data from 1970 to 2000. Additionally, we downloaded elevation data from the Minnesota Geospatial Information Office (MnGeo; https://www.mngeo.state.mn.us/) at a resolution of 30 arc sec (~0.0083°). To refine and improve the ENMs, we downloaded 65 soil composition variables from World Soil Information (ISRIC; https://www.isric.org/) at a resolution of 8 arc sec (~0.00225°). These variables were later adjusted to a resolution of 0.5°. To avoid using highly correlated variables (Dormann et al. 2012), we applied the Spearman coefficient with a threshold of 0.6 in two distinct approaches: one for climate and elevation variables and another for the soil composition variables. For the first, we selected the following variables: elevation, isothermality (bio3), precipitation seasonality (bio15), average monthly precipitation of February (prec_2), and average monthly maximum temperature of October (tmax_10) (Table S2). In the second correlation analysis, the following variables were selected: derived saturated water content (volumetric fraction) teta‐S for depth of 30 cm (AWCtS_M_sl4_250m_ll), depth to bedrock (R horizon) to a maximum of 200 cm (BDRICM_M_250m_ll), absolute depth to bedrock (in cm) (BDTICM_M_250m_ll), clay content (0–2 μm) mass fraction (%) (CLYPPT_M_sl2_250m_ll), soil organic carbon content (fine earth fraction) in g per kg at depth 0.15 m (ORCDRC_M_sl3_250m_ll), and silt content (2–50 μm) mass fraction in % at depth 0.05 m (SLTPPT_M_sl2_250m_ll) (Table S3).

For paleoclimatic reconstructions during the Last Glacial Maximum (LGM), climate data from the Coupled Model Intercomparison Project Phase 3 (CMIP3) were obtained within the CHELSA database (https://chelsa‐climate.org/). We selected three General Circulation Models (GCMs) from independent institutions based on previous ENM analyses: MPI‐ESM‐P (Max Planck Institute for Meteorology), MIROC‐ESM (Japan Agency for Marine‐Earth Science and Technology [JAMSTEC] and Centre for Climate System Research/National Institute for Environmental Studies, Japan), and NCAR CCSM4 (National Center for Atmospheric Research). All climate layers were downloaded at a resolution of 30 arc sec (~0.0083°) and subsequently adjusted to 0.5°.

To model future distributions, we used data from the Coupled Model Intercomparison Project Phase 6 (CMIP6), selecting GCMs that best fit South American climate predictions based on simulations of precipitation and near‐surface air temperature during the historical period (1996–2014) (Dias and Reboita 2021). To avoid the “hot model” problem, where highly sensitive GCMs in CMIP6 overestimate historical temperature trends, we chose GCMs with equilibrium climate sensitivity (ECS) values close to 1.4°C–2.2°C and transient climate response (TCR) values between 2.5°C and 4°C (Sherwood et al. 2020; Hausfather et al. 2022). High TCR values tend to correlate with higher ECS values, as ECS measures the long‐term equilibrium climate response, while TCR reflects a transient state before full climatic adjustment (Tokarska et al. 2020; Zelinka et al. 2020; Hausfather et al. 2022). Based on these criteria, we selected the following three GCMs for future projections: EC‐Earth3 (EC‐Earth consortium), MPI‐ESM1‐2‐LR (Max Planck Institute for Meteorology), and IPSL‐CM6A‐LR (Institut Pierre‐Simon Laplace). We focused on the near‐term period 2021–2040 under two Shared Socioeconomic Pathways (SSPs) to assess the potential distribution of *Dipteryx* species over the next few years. These SSPs represent moderate (SSP3‐7.0; intermediate to SSP5‐8.5, which CO_2_ emissions will double from current levels by 2100 resulting from the absence of additional climate policies and with particularly high non‐CO_2_ emissions) and worse or high emission scenario (SSP5‐8.5; high‐emission scenario, in which CO_2_ emissions are projected to double from current levels by 2050 and with no additional climate policy) (IPCC 2021). We downloaded the netCDF files for the climate series for every 10 years (2021–2030 and 2031–2040) from CHELSA V2.1 (CHELSA Project, https://www.chelsa‐climate.org/) using the *chelsa_cmip6* package (Karger et al. 2023). We then extracted climate variables and constructed raster layers using the R packages *ncdf4* (Pierce 2024) and *raster* (Hijmans et al. 2023).

#### 2.2.2 Model Construction and Analysis

Models for LGM, present, and future times were constructed using *dismo* (Hijmans et al. 2020), *kernlab* (Karatzoglou et al. 2019), *wallace* (Kass et al. 2018), and *randomForest* (Breiman et al. 2022) packages for the R language (R Core Team 2024). Species niches were modeled using five algorithms: presence‐only (BIOCLIM and Domain; Busby 1991; Carpenter et al. 1993), presence–pseudoabsence (SVM; Vapnik 1995), and presence–absence (generalized linear model [GLM] and Random Forest; McCullagh and Nelder 1989; Breiman 2001). Since absences and pseudoabsences were not available, pseudoabsences were randomly selected from cells where no records were found. Our dataset ensures 50% presence and 50% absence, that is, a proportion of 1:1 between presence and pseudoabsence, as indicated for optimal performance of the algorithms SVM and Random Forest and because this proportion does not greatly affect the accuracy of the GLM algorithm (Barbet‐Massin et al. 2012). In order to standardize the models and to avoid confounding comparative interpretation between all 10 species, periods, and future scenarios assessed, we did not define species‐specific accessible areas (M) or apply spatial filters (e.g., geographic rarefaction or environmental thinning). Occurrence data were divided into two subsets, one comprising 70% of presence cells for model calibration and the other containing 30% of presence cells to test model prediction ability. This process was repeated 100 times for each algorithm. Subsequently, a consensus on all geographic distribution predictions was generated. To evaluate model performance, we implemented a cross‐validation approach using 500 replicates (5 algorithm × 100 repetition) per model. For each species, periods, and future scenarios, performance was assessed using the Area Under the Receiver Operating Characteristic Curve (AUC) and the True Skill Statistic (TSS) based on the sensitivity‐specificity method. Only models with AUC ≥ 0.75 and TSS ≥ 0.7 were selected for ensemble predictions (Table S4). Ensemble models were generated using a weighted average approach, where each selected replicate model was standardized using range normalization, and then combined through a weighted mean. The weights were based on the squared TSS values of each replicate, giving more importance to better‐performing models. This general ensemble was calculated for each species using all algorithms that passed the performance threshold, resulting in a final continuous suitability raster per species (consensus map).

The rasters of intersection, as well as gained and lost areas between the present and LGM or future scenarios, were built after binarizing (1 = presence, 0 = absence) the consensus map under the lowest presence threshold (LPT) using the *raster* package (Figures S2 and S3) (Hijmans et al. 2023), which corresponds to the lowest predicted suitability value associated with any known occurrence, ensuring that all training points fall within the predicted presence area (Liu et al. 2005). The area in km^2^ was estimated from the suitable habitat under current and future scenarios or LGM; we used binary raster layers and calculated pixel area with the *area* function from the *raster* package (Hijmans et al. 2023) in R. This function computes pixel area based on the WGS 84 geographic coordinate system (EPSG:4326), accounting for Earth's curvature. To quantify changes in potential distribution area over time, we calculated the percentage difference between the total area predicted as suitable under each past and future scenario relative to the current distribution. The binarized maps were used, and area was computed from raster cells classified as “presence.” Percentage changes were calculated as: (Period/Scenario Area − Present Area)/Present Area × 100. Positive values indicate potential distribution expansion, and negative values indicate potential distribution contraction relative to the present‐day distribution. The uncertainty maps were generated by calculating the standard deviation using the *calc* function from the *raster* package in R (Hijmans et al. 2023). To calculate the uncertainty between the GCMs of each species for LGM and future scenarios, we performed an ensemble of the best models for each of the GCMs using the same methods described above for the general ensemble (Figures [Link], [Link]). Finally, the same methods were applied to calculate the uncertainty between the algorithms of each species for the present day and for each GCM for LGM and future scenarios (Figures [Link], [Link]). Figures, including maps and plots, were constructed using the R packages *ggplot2* (Wickham et al. 2024), *ggspatial* (Dunnington et al. 2023), and *cowplot* (Wilke 2024).

To estimate the potential financial loss lossdue to the near‐term global changes affecting the potential distribution of *Dipteryx* timber species, the total area gained or lost (in km^2^) was extracted from the LPT binarized rasters using the R package *raster* (Hijmans et al. 2023) and converted to hectares (ha). Due to the scarcity of studies analyzing the expected volume per ha for *Dipteryx* timber species, we adopted the estimate of 1.74 m^3^ ha^−1^, which was reported for individuals with a diameter at breast height (DBH) ≥ 50 cm of *D. odorata* from the Floresta Nacional do Tapajós (Pará, Brazil) (Vieira et al. 2021). Finally, to estimate the value per cubic meter of *Dipteryx* timber, we adopted the approximate export price of *cumaru* timber (1000 USD per m^3^; ITTO 2024). This approach assumes a static price and a fixed timber volume and does not account for ecological variability such as forest structure, age, tree density, or taxon‐specific growth patterns across the Neotropics. As such, this projection should be interpreted as an illustrative estimate, aimed at highlighting the potential scale of economic impact due to climate‐driven distribution shifts. Further studies with regionally explicit forest inventory data are necessary for more precise projections. Plots were constructed using the *ggplot2* (Wickham et al. 2024) and *cowplot* (Wilke 2024) R packages.

## 3 Results

**FIGURE 2:**
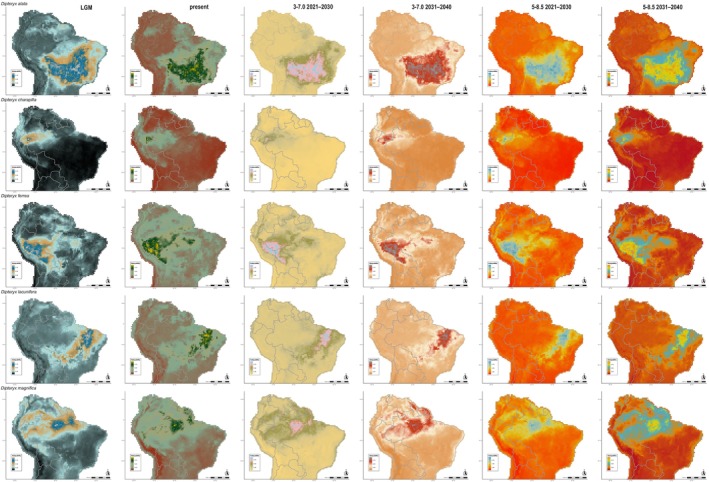
Potential distribution of *D. alata* , *D*. *charapilla*, *D. ferrea* , *D*. *lacunifera*, and *D. magnifica* in Last Glacial Maximum (LGM), currently, and in every 10 years in the near‐term future (2021–2040) under 3–7.0 (moderate) and 5–8.5 (worse scenario) Shared Socioeconomic Pathways (SSPs). The columns represent each period and future scenarios, represented in a distinct scale of colors. The line represents each of the five species.

**FIGURE 3:**
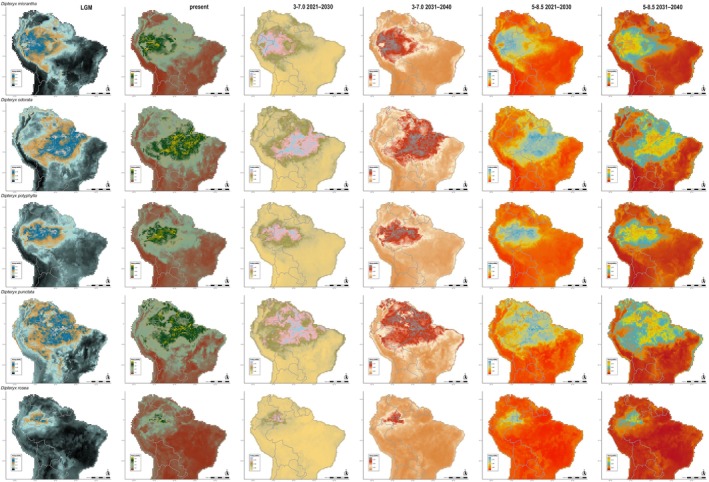
Potential distribution of *D. micrantha* , *D. odorata* , *D*. *polyphylla*, *D. punctata* , and *D. rosea* in the Last Glacial Maximum (LGM), currently, and in every 10 years in the near‐term future (2021–2040) under 3–7.0 (moderate) and 5–8.5 (worse scenario) Shared Socioeconomic Pathways (SSPs). The columns represent each period and future scenarios, represented in a distinct scale of colors. The line represents each of the five species.

**FIGURE 4:**
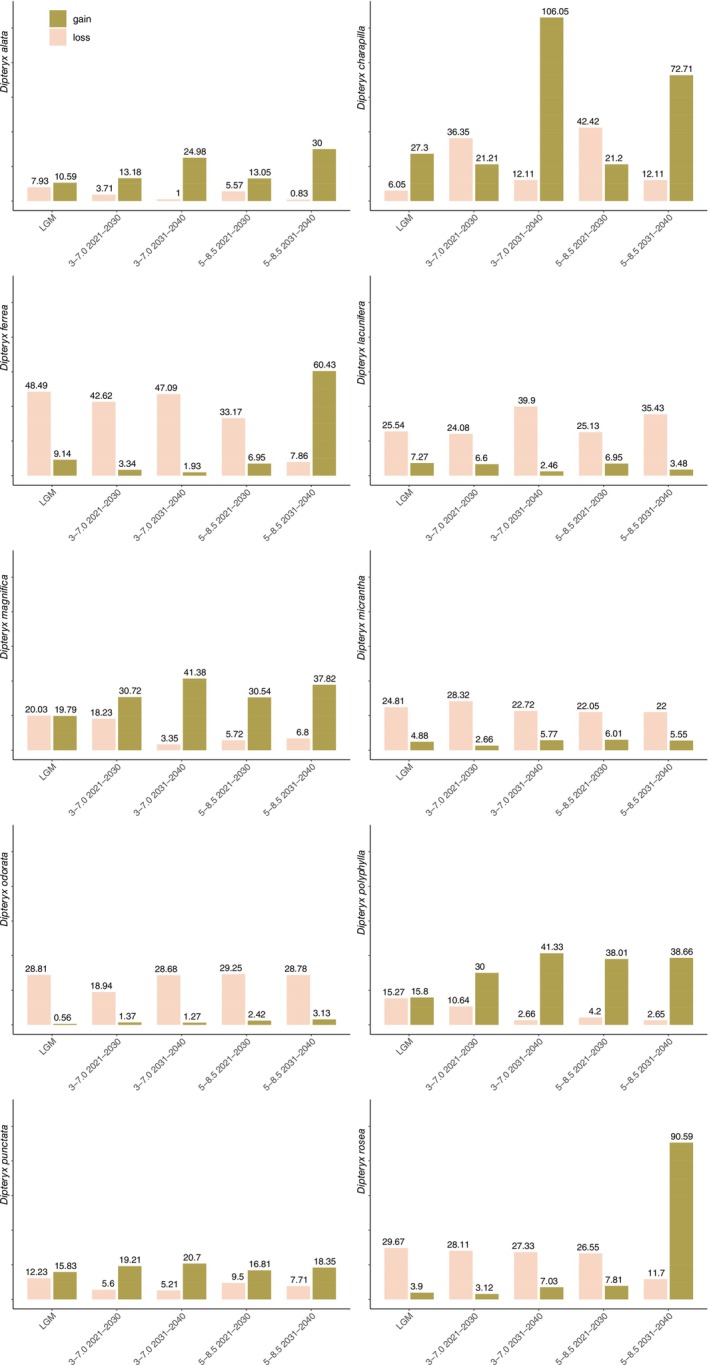
Areas gained and lost (%) in Last Glacial Maximum (LGM) and in every 10 years in near‐term future (2021–2040) under 3–7.0 (moderate) and 5–8.5 (worse scenario) Shared Socioeconomic Pathways (SSPs) relative to the current potential distribution of *Dipteryx* species.

The ENM analysis for the LGM and the next 20 years under future greenhouse gas emission scenarios revealed distributional shifts in all *Dipteryx* species studied when compared with the present (Figures 2, 3, 4, Figures S2 and S3). The patterns of loss and expansion of the occurrence area in response to temperature increases differed between species and biomes (Table 1). For the Cerrado‐inhabiting species *D. alata* , the projections show distinct patterns depending on the emission scenario. Under the moderate‐emissions scenario, the total distribution is projected to expand by 9.47% from today until 2030, and by 23.9% by 2040 (Table 1). Under the high‐emissions scenario, the expansion is 7.48% by 2030 and 29.17% by 2040 (Table 1). During the LGM, the total area of occurrence expanded by 2.66% compared with the present, with potential range extensions toward the central region of the continent (Table 1; Figures 2 and 4, Figure S2). For this species, we verified an increase in the potential distribution toward the Atlantic Forest, Caatinga, and peripheral zones of Amazonia in all scenarios (Figure S2). For *D*. *lacunifera*, a decline in occurrence area in all periods and future scenarios was observed (LGM = −18.27%; SSP3‐7.0: 2021–2030 = −17.48% and 2031–2040 = −37.44%; SSP5‐8.5: 2021–2030 = −18.18% and 2031–2040 = −31.95%). Its distribution is concentrated in the Northeast of Brazil, without any sign of migration from the present distribution during the LGM and future scenarios (Figure 2 and Figure S2). Regarding the Amazonian species, *D. magnifica* suffered a decrease during the LGM (−0.24%) and, along with *D*. *polyphylla* and *D. punctata* , showed gains in the potential area of occurrence in all future scenarios (see Table 1). *Dipteryx rosea* loses its potential area during the LGM and future scenarios, except in SSP5‐8.5 2031–2040 (78.89%). All other Amazonian species, nonetheless, lose their total potential distribution area in every period and future scenario (see Table 1). The area occupied by Amazonian species remained relatively stable in all periods, but an area loss was observed concentrated in the central and peripheral zones of Amazonia (Figure 4, Figures S2 and S3).

**TABLE 1:** Total accumulated in km^2^ (%) by *Dipteryx* species in the Last Glacial Maximum (LGM) and every 10 years in near‐term future (2021–2040) relative to current days.

Species	LGM	3–7.0		5–8.5	
		2021–2030	2031–2040	2021–2030	2031–2040
*D. alata*	2.66%	9.47%	23.98%	7.48%	29.17%
*D*. *charapillla*	21.25%	−15.14%	93.94%	−21.22%	60.6%
*D. ferrea*	−39.35%	−39.28%	−45.16%	−26.22%	52.57%
*D*. *lacunifera*	−18.27%	−17.48%	−37.44%	−18.18%	−31.95%
*D. magnifica*	−0.24%	12.49%	38.03%	24.82%	31.02%
*D. micrantha*	−19.93	−25.66%	−16.95%	−16.04%	−16.45%
*D. odorata*	−28.25%	−17.57%	−27.41%	−26.83%	−25.65%
*D*. *polyphylla*	0.53%	19.36%	38.67%	33.81%	36.01%
*D. punctata*	3.6%	13.61%	15.49%	7.31%	10.64%
*D. rosea*	−25.77%	−24.99%	−20.3%	−18.74%	78.89%

*Note:* The future scenarios were simulated from two Shared Socioeconomic Pathways (SSPs) for two possible future greenhouse gas emissions: SSP3‐7.0 (moderate) and SSP5‐8.5 (worse scenario). Values represent changes in the binarized suitable area under the lowest threshold of presence. Positive values indicate expansion; negative values indicate contraction.

**FIGURE 5:**
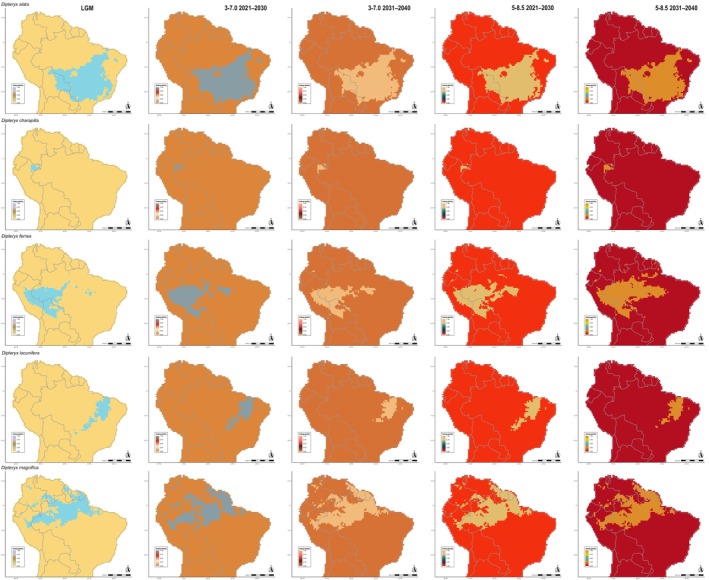
Areas maintained in Last Glacial Maximum (LGM) and in every 10 years in the near‐term future (2021–2040) under 3–7.0 (moderate) and 5–8.5 (worse scenario) Shared Socioeconomic Pathways (SSPs) relative to the current potential distribution of *D. alata* , *D*. *charapilla*, *D. ferrea* , *D*. *lacunifera*, and *D. magnifica* .

**FIGURE 6:**
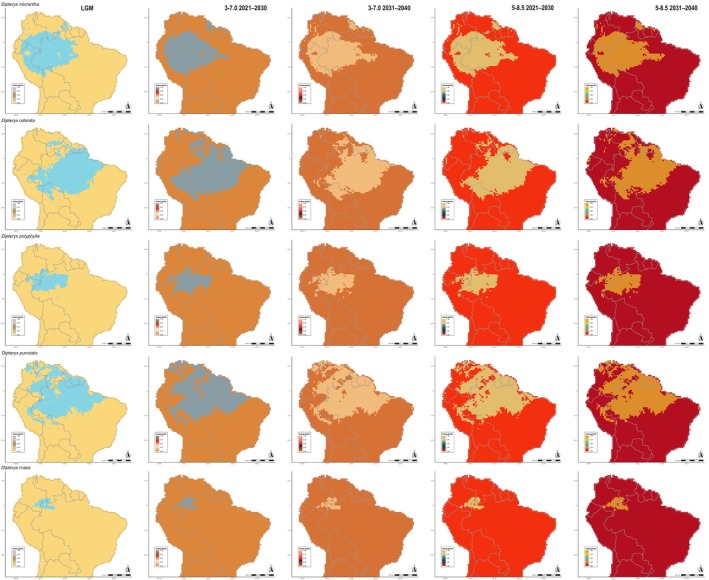
Areas maintained in Last Glacial Maximum (LGM) and in every 10 years in the near‐term future (2021–2040) under 3–7.0 (moderate) and 5–8.5 (worse scenario) Shared Socioeconomic Pathways (SSPs) relative to the current potential distribution of *D. micrantha* , *D. odorata* , *D*. *polyphylla*, *D. punctata* , and *D. rosea* .

The overlap between the current and simulated areas of occurrence during the LGM and future scenarios showed distinct patterns among *Dipteryx* species, but all of them retained at least 50% of the current area of occurrence (Figures 5 and 6; Table 2). *Dipteryx alata* maintained more than 90% of its current area of occurrence in all periods and scenarios (Table 2). During the LGM, *D*. *lacunifera* accounted for 74.45% of the intersected areas, but in both future scenarios, there is a high loss of occurrence area in 2031–2040 (SSP3‐7.0 = 60.10% and SSP5‐8.5 = 64.57%; Figures 2 and 5; Table 2). The Amazonian species retained the largest amount of their area in all periods and scenarios (Figures 5 and 6; Table 2). *Dipteryx charapilla* retained more than 93.94% of its current area during the LGM and 57%–89% in future periods and scenarios (Figures 5 and 6; Table 2). For *D. rosea* , projections indicate that at least 70% of its area was maintained in all periods and scenarios (Figures 5 and 6; Table 2). For *D. ferrea* , the ENM analysis showed that 51.50% of the occurrence area during the LGM coincides with the present. In the worst emission scenarios for the next 20 years, the species is expected to retain an even larger area (2021–2030 = 66.83% and 2031–2040 = 92.14%) (Figure 5; Table 2). For the remaining Amazonian species, the overlap between the present and LGM areas was 79.97% for *D. magnifica* and 87.77% for *D. punctata* . In both future greenhouse gas emission scenarios, these species gained area during the studied periods (see Figures 5 and 6; Table 2). Indeed, they remain relatively stable among LGM and future scenarios, having maintained at least 70% of their current area.

**TABLE 2:** Percentage potential distribution area in km^2^ maintained in the Last Glacial Maximum (LGM) and every 10 years in the near‐term future (2021–2040) relative to current days.

Species	LGM	3–7.0		5–8.5	
		2021–2030	2031–2040	2021–2030	2031–2040
*D. alata*	92.06%	96.29%	99%	94.43%	99.17%
*D*. *charapilla*	93.94%	75%	87.89%	57.58%	87.89%
*D. ferrea*	51.50%	57.38%	52.91%	66.83%	92.14%
*D*. *lacunifera*	74.45%	75.91%	60.10%	74.87%	64.57%
*D. magnifica*	79.97%	81.77%	96.65%	94.28%	93.2%
*D. micrantha*	75.19%	71.68%	77.28%	77.95%	78%
*D. odorata*	71.19%	81.06%	71.32%	70.74%	71.21%
*D*. *polyphylla*	84.73%	89.36%	97.34%	95.8%	97.34%
*D. punctata*	87.77%	94.39%	94.79%	90.50%	92.29%
*D. rosea*	70.33%	71.88%	72.67%	73.45%	88.29%

*Note:* The future scenarios were simulated using two Shared Socioeconomic Pathways (SSPs) for two possible future greenhouse gas emissions: 5–8.5 (worse scenario) and 3–7.0 (moderate). Values were calculated based on the lowest threshold of presence.

**FIGURE 7:**
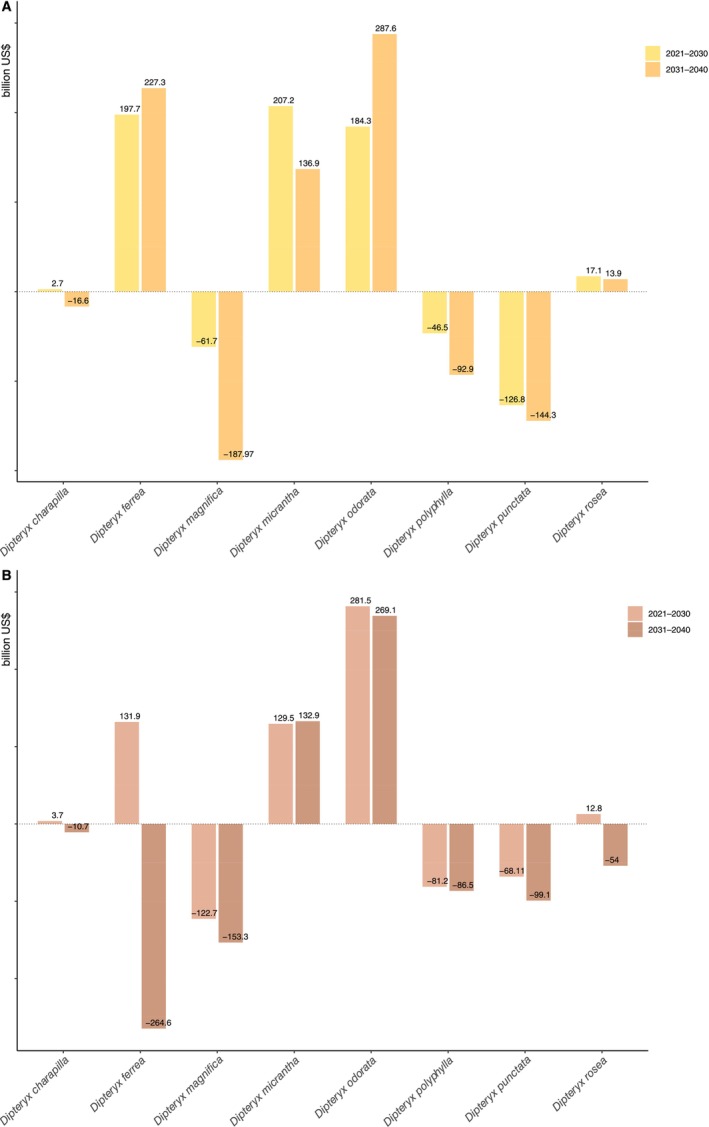
Amount in billions of dollars that will be potentially lost in the near‐term future (2021–2040) under (A) 3–7.0 (moderate) and (B) 5–8.5 (worse scenario) Shared Socioeconomic Pathways (SSPs) due to decreasing potential distribution of *Dipteryx* timber species. The sum of values represents the total amount of money impacted by the lost area of *Dipteryx* timber species (*cumaru*): US$ 597.93 billion dollars (moderate scenario) and US$ 21.19 billion dollars (worse scenario). The negative values (−) are relative to the species that presented a gain of potential distribution during future scenarios.

When analyzing the economic impact of the potential loss of *Dipteryx* timber species habitat in near‐term future scenarios, a total loss of US$ 597.93 billion is estimated in the moderate scenario and US$ 21.19 billion in the worst scenario (see Figure 7 for individual values per species).

## 4 Discussion

### 4.1 Temporal Analysis of *Dipteryx* Species Reveals Stability in Amazonian Habitats Under Near‐Term Future Climate Change

The distribution of *Dipteryx* Amazonian species over time and across different greenhouse gas emission scenarios was generally stable, with a large portion of the current distribution area overlapping with both LGM and future scenario predictions (Figures 2, 3, 4, 5, 6; Figures S2 and S3). However, species primarily distributed in Central and Eastern Amazonia, *D. odorata* and *D. punctata* , tended to lose area in the peripheral zones of the forest, particularly in ecotones between Amazonia and Cerrado, as well as in Amazonian savannas (Figures 2, 3, 4; Figures S2 and S3). Indeed, the Amazonian rainforest has persisted since the beginning of the Cenozoic (~65 Mya), withstanding geological weathering, shifts in drainage direction and sediment deposition (~7 Mya), Pleistocene (~21 kya), and Holocene (~6 kya) climatic changes, and human occupation (Colinvaux et al. 1996; Hoorn et al. 2010; Wang et al. 2017; Clement et al. 2021; Kukla et al. 2021; Flores et al. 2024). Despite its resilience and stability, records indicate localized expansions of savanna‐like vegetation during dry periods in peripheral forest areas, particularly in seasonal climates during the LGM and regions affected by human fires in the Holocene (Mayle and Power 2008; Flores et al. 2024; Kelley et al. 2024). Furthermore, since the 1980s, rainfall has decreased significantly in both peripheral and Central Amazonia (Flores et al. 2024). In the future, intensified droughts could significantly impact most seasonal regions of the forest, which already experience water stress during drier months (Flores et al. 2024), as verified for *D. odorata* (Figures 2, 3, 4; Figures S2 and S3; Table 1). Conversely, the fertile soils of Western Amazonia are expected to show greater resilience due to increased rainfall and photosynthetic rates, facilitated by atmospheric CO_2_ fertilization (Ainsworth and Long 2005; Quesada et al. 2012; Ellsworth et al. 2017; Flores et al. 2024). This resilience may lead to an expansion in the total distribution area and facilitate migration of *D*. *charapilla*, *D. ferrea* , *D. micrantha* , and *D*. *polyphylla* (Figures 2, 3, 4; Figures S2 and S3; Table 1). However, *Dipteryx* species' habitat specialized in Western Amazonia could also lose up to ~45% of their total area in near‐term future scenarios. Furthermore, due to their confined distribution, areas of overlap between present, LGM, and near‐term future distributions may be overestimated for these species compared to widely distributed ones (Figures 2, 3, 4, 5, 6; Tables 1 and 2; Junk et al. 2011; Wittmann et al. 2013). This is evident for the Western Amazonian confined *D*. *charapilla* (from *várzeas* of upper Rio Amazonas in Peru and Brazil) and *D. rosea* (from *igapós* of upper Rio Negro in Brazil, Colombia, and Venezuela) (Figure 1 and Figure S1; Carvalho, Cardoso, and Lima 2024). Thus, we believe that further finest‐scale investigations are necessary to assess the resilience locally, mainly for specific Amazonian habitats (e.g., *campina*, *campinara*, *igapó*, and *várzea*) under different global climate change scenarios.

Over the next 20 years, the distribution of *D. alata* in the Cerrado is expected to increase under the worst scenario (Figure 2 and Figure S2; Table 1). The future expansion of *D. alata* may be linked to increasing temperatures, prolonged droughts, and more flammable vegetation predicted for South America (Alvarez et al. 2021; IPCC 2021; Almazroui et al. 2024; Feron et al. 2024). Our predictions do not indicate a migration of *D. alata* into the interior of Amazonia, but rather a potential distribution expansion toward peripheral ecotone zones in Central and Southeastern Amazonia (Figure 2 and Figure S2). This result aligns with the hypothesis that Amazonia will remain resilient and resist widespread ruderalization until at least 2040 (IPCC 2021; Flores et al. 2024; Shirai et al. 2024). It is important to emphasize that the spatial–temporal projections presented here assume climatic niche conservatism and that species can colonize all climatically suitable areas (Araújo and Guisan 2006; Peterson and Soberón 2012). Thus, they may not always reflect the actual distribution of the species, particularly in fragmented landscapes such as the Amazonia and Cerrado. Indeed, anthropogenic pressures, including recurrent fires, rising temperatures, increased drought frequency, and deforestation, could weaken this resilience, potentially transforming not only ecotones, but even remote and internal forest areas into more open, ruderal vegetation (IPCC 2021; Flores et al. 2024).

In the past 21 kya, during the LGM, *D. alata* gained some potential distribution (2.66%) and shifted toward current ecotones between the Cerrado and northernmost forests, such as Amazonia, Atlantic Forest, and SDTF, coinciding with drier conditions (Figure 2 and Figure S2). At that time, the southern and southwestern regions of Brazil experienced relatively high humidity and low temperatures, likely forming corridors connecting the Eastern Andes and Atlantic Forest through the drier Cerrado and Chaco (Morley 2000; Cruz et al. 2005; Berman et al. 2016; Trujillo‐Arias et al. 2017; Pinaya et al. 2024). These corridors, known as the Central South American Connectivity and Cerrado‐Chaco connections, extend between 13° and 30° S, with increasingly harsh winters toward the south (Berman et al. 2016; Trujillo‐Arias et al. 2017; Pinaya et al. 2024). Areas with harsh winters correspond to where *D. alata* lost distribution during the LGM compared to present days (Figure 2 and Figure S2).

The spatial–temporal simulations indicated that *D*. *lacunifera* is particularly sensitive to climate change, losing habitat under both drier, cooler LGM conditions and drier, warmer future conditions (Figure 2 and Figure S2). Indeed, the Caatinga vegetation is regarded as among the most vulnerable seasonally dry tropical forests of the world due to future climate change (Marengo et al. 2016; Santos et al. 2014; Seddon et al. 2016). The Caatinga‐endemic *D*. *lacunifera* from the states of Maranhão, Piauí, and Bahia occurs exclusively along watercourses and in the most humid habitats, typically associated with sandy soils or seasonally flooded depressions, that is, environments that mitigate the harsher conditions of the biome (Figure S1; Queiroz et al. 2017; Carvalho, Cardoso, and Lima 2024; Carvalho, Lima, et al. 2024). By 2070–2099, habitat loss in the Caatinga is expected to be extensive, with suitable areas for endemic species likely becoming restricted to regions near the northeastern Atlantic Forest coast (Silva et al. 2019). However, our models do not suggest migration of *D*. *lacunifera* toward the coast by 2040, probably because of its aforementioned ecological preferences. Instead, we observed a habitat loss of up to ~30% to 40% (Figures 2 and 4, Figure S2; Table 1). Due to its reliance on humid habitats in a narrow Caatinga region, *D*. *lacunifera* appears highly vulnerable to future desertification in northeastern South America (Antongiovanni et al. 2018). This is particularly concerning given that approximately 50% of the original Caatinga has already been lost due to human activities such as settlement expansion, fuelwood extraction, livestock farming, and soybean cultivation, leaving fragmented landscapes with patches smaller than 500 ha (Ribeiro et al. 2015; Marinho et al. 2016; Antongiovanni et al. 2018).

### 4.2 The Role of *Dipteryx* Species in the Economy and the Impact of Deforestation During the Near‐Term Future Climate Change

Species of *Dipteryx* are recognized for their economic importance, whether for cosmetics, food, or timber. Due to their hard and durable wood, they have been appreciated as a high‐quality substitute for *ipê* species (*Handroanthus* spp.) in the timber trade (e.g., https://www.ipeoutlet.com/cumaru‐alternative‐to‐ipe/). Indeed, the use of *cumaru* timber has increased substantially, accounting for nearly half of all wood exported from Peru between 2012 and 2020, mainly to China, Europe, and the United States (SERFOR 2017; Castro et al. 2021). In Brazil, logging of these species increased by 55% between 2012 and 2017, even as total wood production declined by 22% in the same period (IBAMA 2019). Although our economic projection is based on a simplified model using static prices and average timber volumes and this estimate still needs further analysis, a large‐scale timber trade could be largely affected by future climate change, with projected economic losses estimated at US$ 597.93 billion under a moderate scenario and US$ 21.19 billion under a worst‐case scenario due to the reduction of suitable areas for *Dipteryx* timber species (Figure 7).

Beyond economic losses, these projections raise serious concerns for Amazonian biodiversity conservation. Large‐scale timber harvesting and trade are directly linked to deforestation and habitat loss, even in near‐term scenarios (Putzel 2009). In addition, since all Amazonian species are potentially traded under the name of the more widely distributed *D. odorata* (and its synonyms or simply *cumaru*), species with restricted distributions are particularly threatened under indiscriminate exploitation. For example, despite *D*. *charapilla* and *D. rosea* showing resilience and even a gain of potential suitable area in worse future scenarios, they have a restricted area in the western Amazonian region (Figures 2, 3, 4, Figures S2 and S3; Table 1). Although the overall potential distribution of Amazonian *Dipteryx* species appears stable in near‐term projections (Figures 5 and 6; Table 2), interactions among multiple disturbances, such as increasingly hotter temperatures, frequent extreme droughts, and forest fires, could accelerate ecosystem shifts toward ruderal vegetation by 2050, even in remote areas of Amazonia (Stropp et al. 2020; IPCC 2021; Flores et al. 2024; Shirai et al. 2024). The conservation efforts for *Dipteryx* species should be coordinated through a multiple‐country Amazon policy agreement. However, Brazil calls attention as a country with high levels of Amazonian deforestation and is also home to major infrastructure projects such as the Carajás mining complex, Belo Monte hydropower plant, and the Trans‐Amazonian Highway. These large‐scale initiatives are closely tied to deforestation expansion from the peripheries (“deforestation arc”) into the interior of Amazonia (Stropp et al. 2020; Brasil 2023; Stegmann et al. 2024).

Non‐timber products derived from *Dipteryx* species also represent a considerable source of income for Indigenous and traditional communities, supporting socio‐economic development and cultural heritage in South America (Marshal et al. 2006). In 2019, the production of *D. odorata* and *D. punctata* seeds in Brazil generated approximately R$ 3 million (US$ 510,000), concentrated in the states of Amazonas and Pará, which produced 33 and 92 tons of seeds, respectively (IBGE 2023). Meanwhile, *D. alata* seeds, commercialized as baru nuts from Cerrado, have gained international attention as an increasingly trendy superfood (Sano et al. 1999; Flynn 2018). In 2017, 110 tons of baru nuts were produced in Brazil, generating R$ 532,000 (approximately US$ 90,000) (IBGE 2023). However, if carried out unsustainably, seed exploitation of *D. alata* , *D. odorata* , and *D. punctata* , whether for food or cosmetics, could threaten their genetic diversity (e.g., Guimarães et al. 2019; Honorio Coronado et al. 2020). While the impacts of future climate change, timber trade, and habitat conversion on these species have been studied, the role of seed harvesting in population decline remains poorly understood (Herrero‐Jáuregui et al. 2012). Additionally, further genetic studies across remaining *Dipteryx* species are also needed to investigate how wood and seed exploitation may impact the genus as a whole. Without effective government policies to protect Amazonia and Cerrado biomes, reduction of greenhouse gas emissions, and mitigation of deforestation, future climate change could jeopardize economic activities that support traditional communities.

### 4.3 *Dipteryx* Preference for Humid Habitats Underscores the Need for Healthy Forests to Conserve Its Amazonian Species

The spatial–temporal diversification of Dipterygeae, the clade to which *Dipteryx* belongs, is closely linked to the transformation of cratonic Amazonia into the Andean‐dominated landscapes of today (Hoorn et al. 2010, 2022; Carvalho, Lima, et al. 2024). *Dipteryx* diversification has revealed a long evolutionary history of ecological predilection and persistence in the Amazonian rainforest (~18 Mya), with only four known dispersal events into other regions (Carvalho, Lima, et al. 2024). The species that originated from these extra‐Amazonian dispersal events are now found in other rainforests (e.g., Chocó and Central American humid forests, and the Atlantic Forest) or in less extreme environments along watercourses in the Cerrado and Caatinga ( *D. alata* and *D*. *lacunifera*, respectively) (Carvalho, Lima, et al. 2024). These findings suggest a strong signature of niche conservatism, indicating that despite their colonization of new geographic regions, *Dipteryx* species continue to prefer environmental conditions similar to those of Amazonia (Donoghue and Edwards 2014; Carvalho, Lima, et al. 2024). Thus, humid conditions are important for their survival and conservation, regardless of habitat. For instance, during the LGM, *D. alata* occupied ancient humid corridors, which were wetter than Cerrado today and connected forests from the Andes to the Atlantic Forest. This suggests that humidity was not a limiting factor for *D. alata* during this period (Figure 2 and Figure S2; see also Section 4.1; Berman et al. 2016; Trujillo‐Arias et al. 2017; Pinaya et al. 2024). Similarly, *D*. *lacunifera* inhabits relatively humid Caatinga habitats, often associated with sandy soils or seasonally flooded depressions, and is highly sensitive to drier conditions, such as those observed during the LGM and in future greenhouse gas emission scenarios (Figure 2 and Figure S2; Table 1; Queiroz et al. 2017; Carvalho, Lima, et al. 2024).

Alarmingly, the strong preference for humid habitats observed in Amazonian *Dipteryx* species suggests that healthy forests are essential for their survival. However, the central and peripheral Amazonian regions are experiencing increasing dryness, with ca. 38% of the forest already affected by human disturbance, including logging, edge effects, understory fires, and recurrent extreme droughts (Bullock et al. 2020; Marengo et al. 2022; Lapola et al. 2023; Flores et al. 2024). Despite the apparent stability observed in the distribution of *Dipteryx* species over the next two decades based on climate and soil variables (Figures 2, 3, 4, 5, 6; Tables 1 and 2), the combined pressures of logging and deforestation disturbances could push these species toward extinction.

While our models project future distributions based on suitable environmental conditions, they inherently assume no dispersal limitations for the species, meaning that taxa are presumed to reach all climatically suitable areas. In practice, barriers such as habitat fragmentation, geographic distance, or limited seed dispersal mechanisms may prevent *Dipteryx* species from occupying these predicted future habitats. This assumption, together with that of niche conservatism, may lead to optimistic projections, especially under future climate changes and rapidly changing landscapes (Araújo and Guisan 2006; Peterson and Soberón 2012). Additionally, for regions with strong climatic shifts, such as the Caatinga, rapid changes in precipitation and temperature could drive local adaptations or even niche evolution, which could reduce the accuracy of predictions. To prevent the loss of *Dipteryx* species and the disruption of an economic activity that benefits local traditional communities, urgent measures are needed to curb deforestation, regulate local and international logging, and reinforce the need to stop greenhouse gas emissions (IPCC 2021; Brasil 2023; Flores et al. 2024).

## 5 Conclusions

The papilionoid legume species of the genus *Dipteryx* (e.g., *baru*, *cumaru*, and *shihuahuacu*) are recognized for their economic potential, both as sources of high‐quality timber and for their non‐timber products, which provide income for many traditional communities across South America. In this study, we used ecological niche modeling to assess the potential distribution of 10 *Dipteryx* species under future global warming scenarios. We analyzed greenhouse gas emissions projections from GCMs under two scenarios: SSP3‐7.0 (moderate) and SSP5‐8.5 (worst‐case scenario). Additionally, we incorporated elevation and soil variables to refine predictions. To further evaluate the resilience of *Dipteryx* species across different climatic periods, we also assessed their potential distribution during the LGM. The key findings of this study are as follows:

- The distribution of Amazonian *Dipteryx* species remained stable during both the LGM and near‐term future greenhouse gas emission scenarios, aligning with expectations of Amazonian forest resilience. However, species with restricted distribution, even in high‐resilience and high‐precipitation zones, showed sensitivity to global warming.
- In the near future, the potential distribution of the Cerrado‐inhabiting species *D. alata* is expected to expand toward ecotonal areas between the Cerrado and humid forests. This expansion will likely be driven by rising temperatures, prolonged drought periods, and more flammable savanna‐like vegetation that is expected to expand over the next 20 years.
- Just like the predicted habitat loss during the LGM, *Dipteryx lacunifera* is projected to lose up to ~30% to 40% of its potential distribution toward Caatinga in the near‐term future, mainly due to drier conditions. Conservation of the species is particularly alarming, given the ongoing habitat loss driven by human settlement, fuelwood extraction, and livestock farming.
- Despite the relative stability of Amazonian *Dipteryx* species, logging and deforestation pressures within Amazonian habitats pose significant threats to their survival. Given their distribution across the Amazonian countries and economic importance, coordinated conservation efforts and policy agreements among multiple Amazonian nations are needed. Such regulatory actions could avoid habitat degradation and its detrimental consequences not only for the economy and biodiversity, but also for Indigenous and traditional communities that rely on these species.
- *Dipteryx* species have a strong preference for humid environments, particularly Amazonian species, which depend on the maintenance of healthy forest ecosystems for their survival and conservation.

While this study provides valuable insights into the past (LGM) and near‐term future distribution of *Dipteryx* species, further assessments of potential distribution for mid‐term (2041–2060) and long‐term (2081–2100) scenarios are necessary. Additionally, it remains essential to evaluate near‐term future projections at a more localized and fine scale focused on the potential distribution of each *Dipteryx* species. This approach will be essential to understand how climate change may affect distinct South American biomes, as well as each Amazonian environment, such as savannas, *campinaranas*, *várzeas*, and *igapós*. Such analyses will help refine our understanding of the impacts of climate change on *Dipteryx* species populations, aiding in the development of conservation strategies to prevent species extinctions and better anticipate biome shifts across South America.

## Author Contributions

**Catarina S. Carvalho:** conceptualization (equal), data curation (equal), formal analysis (equal), funding acquisition (equal), investigation (equal), methodology (equal), project administration (equal), writing – original draft (lead), writing – review and editing (equal). **Raquel Moura Machado:** writing – review and editing (equal). **Maristerra R. Lemes:** supervision (equal), writing – review and editing (equal). **Domingos Cardoso:** funding acquisition (equal), supervision (equal), writing – review and editing (equal).

## Conflicts of Interest

The authors declare no conflicts of interest.

## Supporting information


**Data S1:** ece372105‐sup‐0001‐algorithms.R.

**Data S2:** ece372105‐sup‐0002‐binarization.R.

**Data S3:** ece372105‐sup‐0003‐Chelsa‐CMP6‐extracnetcdf.R.

**Data S4:** ece372105‐sup‐0004‐ensemble.R.

**Data S5:** ece372105‐sup‐0005‐futureLGMvspresent.R.

**Figure S1:** Geographical occurrence of the South American *Dipteryx* species overlapping the estimated potential distribution for the present.

**Figure S2:** Maps of areas gained and lost in Last Glacial Maximum (LGM) and in every 10 years in near‐term future (2021–2040) under 3–7.0 (moderate) and 5–8.5 (worse scenario) Shared Socioeconomic Pathways (SSPs) relative to the current potential distribution of *D. alata* , *D*. *charapilla*, *D. ferrea* , *D*. *lacunifera*, and *D. magnifica* demonstrating the pattern of migration of *Dipteryx* species.

**Figure S3:** Maps of areas gained and lost in Last Glacial Maximum (LGM) and in every 10 years in near‐term future (2021–2040) under 3–7.0 (moderate) and 5–8.5 (worse scenario) Shared Socioeconomic Pathways (SSPs) relative to the current potential distribution of *D. micrantha* , *D. odorata* , *D*. *polyphylla*, *D. punctata* , and *D. rosea* to demonstrate the pattern of migration of *Dipteryx* species.

**Figure S4:** Maps of uncertainty between the General Circulation Models (GCMs) MPI‐ESM‐P, MIROC‐ESM, and NCAR CCSM4 of Last Glacial Maximum (LGM) and between EC‐Earth3, MPI‐ESM1‐2‐LR, and IPSL‐CM6A‐LR for the near‐term future (2021–2040) assessed in every 10 years under 3–7.0 (moderate) and 5–8.5 (worse scenario) Shared Socioeconomic Pathways (SSPs) relative to the *D. alata* , *D*. *charapilla*, *D. ferrea* , *D*. *lacunifera*, and *D. magnifica* .

**Figure S5:** Maps of uncertainty between the General Circulation Models (GCMs) MPI‐ESM‐P, MIROC‐ESM, and NCAR CCSM4 of Last Glacial Maximum (LGM) and between EC‐Earth3, MPI‐ESM1‐2‐LR, and IPSL‐CM6A‐LR for the near‐term future (2021–2040) assessed in every 10 years under 3–7.0 (moderate) and 5–8.5 (worse scenario) Shared Socioeconomic Pathways (SSPs) relative to the *D. micrantha* , *D. odorata* , *D*. *polyphylla*, *D. punctata* , and *D. rosea* .

**Figure S6:** Maps of uncertainty between the algorithm presence‐only (BIOCLIM and Domain), presence–pseudoabsence (SVM), and presence–absence (generalized linear model [GLM] and Random Forest) relative to *D. alata* . (A) Present days. (B–D) Last Glacial Maximum (LGM) General Circulation Models (GCMs): MIROC‐ESM (B), MPI‐ESM‐P (C), and NCAR CCSM4 (D). (E–P) Near‐term future GCMs under 3–7.0 (moderate) and 5–8.5 (worse scenario) Shared Socioeconomic Pathways (SSPs): EC‐Earth3, IPSL‐CM6A‐LR, and MPI‐ESM1‐2‐LR.

**Figure S7:** Maps of uncertainty between the algorithm presence‐only (BIOCLIM and Domain), presence–pseudoabsence (SVM), and presence–absence (generalized linear model [GLM] and Random Forest) relative to *D*. *charapilla*. (A) Present days. (B–D) Last Glacial Maximum (LGM) General Circulation Models (GCMs): MIROC‐ESM (B), MPI‐ESM‐P (C), and NCAR CCSM4 (D). (E–P) Near‐term future GCMs under 3–7.0 (moderate) and 5–8.5 (worse scenario) Shared Socioeconomic Pathways (SSPs): EC‐Earth3, IPSL‐CM6A‐LR, and MPI‐ESM1‐2‐LR.

**Figure S8:** Maps of uncertainty between the algorithm presence‐only (BIOCLIM and Domain), presence–pseudoabsence (SVM), and presence–absence (generalized linear model [GLM] and Random Forest) relative to *D. ferrea* . (A) Present days. (B–D) Last Glacial Maximum (LGM) General Circulation Models (GCMs): MIROC‐ESM (B), MPI‐ESM‐P (C), and NCAR CCSM4 (D). (E–P) Near‐term future GCMs under 3–7.0 (moderate) and 5–8.5 (worse scenario) Shared Socioeconomic Pathways (SSPs): EC‐Earth3, IPSL‐CM6A‐LR, and MPI‐ESM1‐2‐LR.

**Figure S9:** Maps of uncertainty between the algorithm presence‐only (BIOCLIM and Domain), presence–pseudoabsence (SVM), and presence–absence (generalized linear model [GLM] and Random Forest) relative to *D*. *lacunifera*. (A) Present days. (B–D) Last Glacial Maximum (LGM) General Circulation Models (GCMs): MIROC‐ESM (B), MPI‐ESM‐P (C), and NCAR CCSM4 (D). (E–P) Near‐term future GCMs under 3–7.0 (moderate) and 5–8.5 (worse scenario) Shared Socioeconomic Pathways (SSPs): EC‐Earth3, IPSL‐CM6A‐LR, and MPI‐ESM1‐2‐LR.

**Figure S10:** Maps of uncertainty between the algorithm presence‐only (BIOCLIM and Domain), presence–pseudoabsence (SVM), and presence–absence (generalized linear model [GLM] and Random Forest) relative to *D. magnifica* . (A) Present days. (B–D) Last Glacial Maximum (LGM) General Circulation Models (GCMs): MIROC‐ESM (B), MPI‐ESM‐P (C), and NCAR CCSM4 (D). (E–P) Near‐term future GCMs under 3–7.0 (moderate) and 5–8.5 (worse scenario) Shared Socioeconomic Pathways (SSPs): EC‐Earth3, IPSL‐CM6A‐LR, and MPI‐ESM1‐2‐LR.

**Figure S11:** Maps of uncertainty between the algorithm presence‐only (BIOCLIM and Domain), presence–pseudoabsence (SVM), and presence–absence (generalized linear model [GLM] and Random Forest) relative to *D. micrantha* . (A) Present days. (B–D) Last Glacial Maximum (LGM) General Circulation Models (GCMs): MIROC‐ESM (B), MPI‐ESM‐P (C), and NCAR CCSM4 (D). (E–P) Near‐term future GCMs under 3–7.0 (moderate) and 5–8.5 (worse scenario) Shared Socioeconomic Pathways (SSPs): EC‐Earth3, IPSL‐CM6A‐LR, and MPI‐ESM1‐2‐LR.

**Figure S12:** Maps of uncertainty between the algorithm presence‐only (BIOCLIM and Domain), presence–pseudoabsence (SVM), and presence–absence (generalized linear model [GLM] and Random Forest) relative to *D. odorata* . (A) Present days. (B–D) Last Glacial Maximum (LGM) General Circulation Models (GCMs): MIROC‐ESM (B), MPI‐ESM‐P (C), and NCAR CCSM4 (D). (E–P) Near‐term future GCMs under 3–7.0 (moderate) and 5–8.5 (worse scenario) Shared Socioeconomic Pathways (SSPs): EC‐Earth3, IPSL‐CM6A‐LR, and MPI‐ESM1‐2‐LR.

**Figure S13:** Maps of uncertainty between the algorithm presence‐only (BIOCLIM and Domain), presence–pseudoabsence (SVM), and presence–absence (generalized linear model [GLM] and Random Forest) relative to *D*. *polyphylla*. (A) Present days. (B–D) Last Glacial Maximum (LGM) General Circulation Models (GCMs): MIROC‐ESM (B), MPI‐ESM‐P (C), and NCAR CCSM4 (D). (E–P) Near‐term future GCMs under 3–7.0 (moderate) and 5–8.5 (worse scenario) Shared Socioeconomic Pathways (SSPs): EC‐Earth3, IPSL‐CM6A‐LR, and MPI‐ESM1‐2‐LR.

**Figure S14:** Maps of uncertainty between the algorithm presence‐only (BIOCLIM and Domain), presence–pseudoabsence (SVM), and presence–absence (generalized linear model [GLM] and Random Forest) relative to *D. punctata* . (A) Present days. (B–D) Last Glacial Maximum (LGM) General Circulation Models (GCMs): MIROC‐ESM (B), MPI‐ESM‐P (C), and NCAR CCSM4 (D). (E–P) Near‐term future GCMs under 3–7.0 (moderate) and 5–8.5 (worse scenario) Shared Socioeconomic Pathways (SSPs): EC‐Earth3, IPSL‐CM6A‐LR, and MPI‐ESM1‐2‐LR.

**Figure S15:** Maps of uncertainty between the algorithm presence‐only (BIOCLIM and Domain), presence–pseudoabsence (SVM), and presence–absence (generalized linear model [GLM] and Random Forest) relative to *D. rosea* . (A) Present days. (B–D) Last Glacial Maximum (LGM) General Circulation Models (GCMs): MIROC‐ESM (B), MPI‐ESM‐P (C), and NCAR CCSM4 (D). (E–P) Near‐term future GCMs under 3–7.0 (moderate) and 5–8.5 (worse scenario) Shared Socioeconomic Pathways (SSPs): EC‐Earth3, IPSL‐CM6A‐LR, and MPI‐ESM1‐2‐LR.

**Table S1:** Occurrence data from the studied species of *Dipteryx*.

**Table S2:** Spearman coefficient results to the 18 climate variables downloaded from WorldClim 2 and elevation (Minnesota Geospatial Information Office; MnGeo).

**Table S3:** Spearman coefficient results to the 65 soil composition variables downloaded from World Soil Information (ISRIC).

**Table S4:** Model evaluation metrics with AUC and TSS values.

## Data Availability

All the data are available in the published version of the manuscript.
