# Supplementary figures and images for: Ecological Niche Modeling Predicts Alarming Impacts of Global Climate Change on Economically Important Neotropical Trees
Source: Ecol Evol. 2025 Sep 23;15(9):e72105. doi: 10.1002/ece3.72105 (PMC12457732; doi:10.1002/ece3.72105)

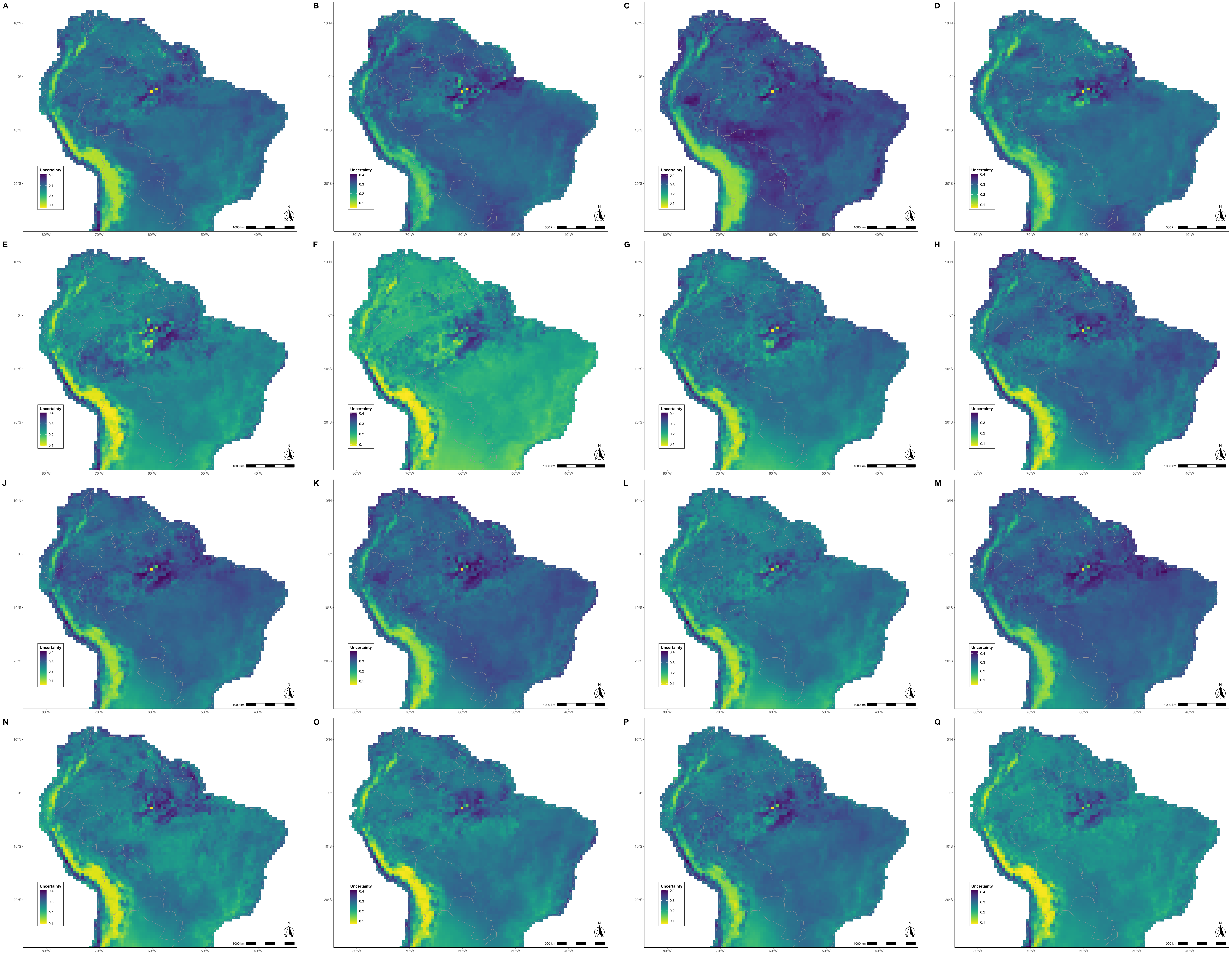

Supplement: Supplementary file 6 — Figure S1: Geographical occurrence of the South American Dipteryx species overlapping the estimated potential distribution for the present. [file ECE3-15-e72105-s016.pdf]

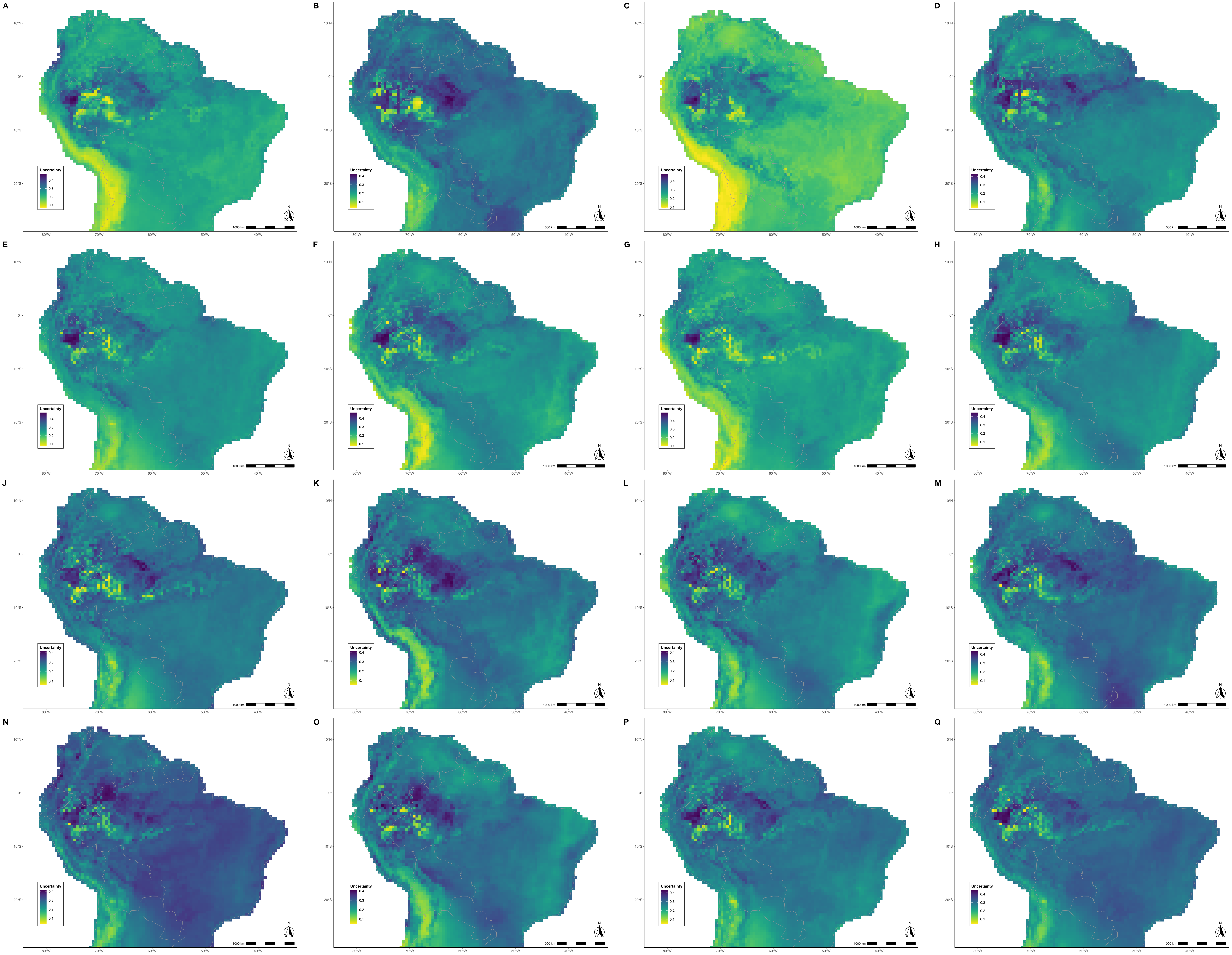

Supplement: Supplementary file 7 — Figure S2: Maps of areas gained and lost in Last Glacial Maximum (LGM) and in every 10 years in near‐term future (2021–2040) under 3–7.0 (moderate) and 5–8.5 (worse scenario) Shared Socioeconomic Pathways (SSPs) relative to the current potential distribution of D. alata , D. charapilla, D. ferrea , D. lacunifera, and D. magnifica demonstrating the pattern of migration of Dipteryx species. [file ECE3-15-e72105-s009.pdf]

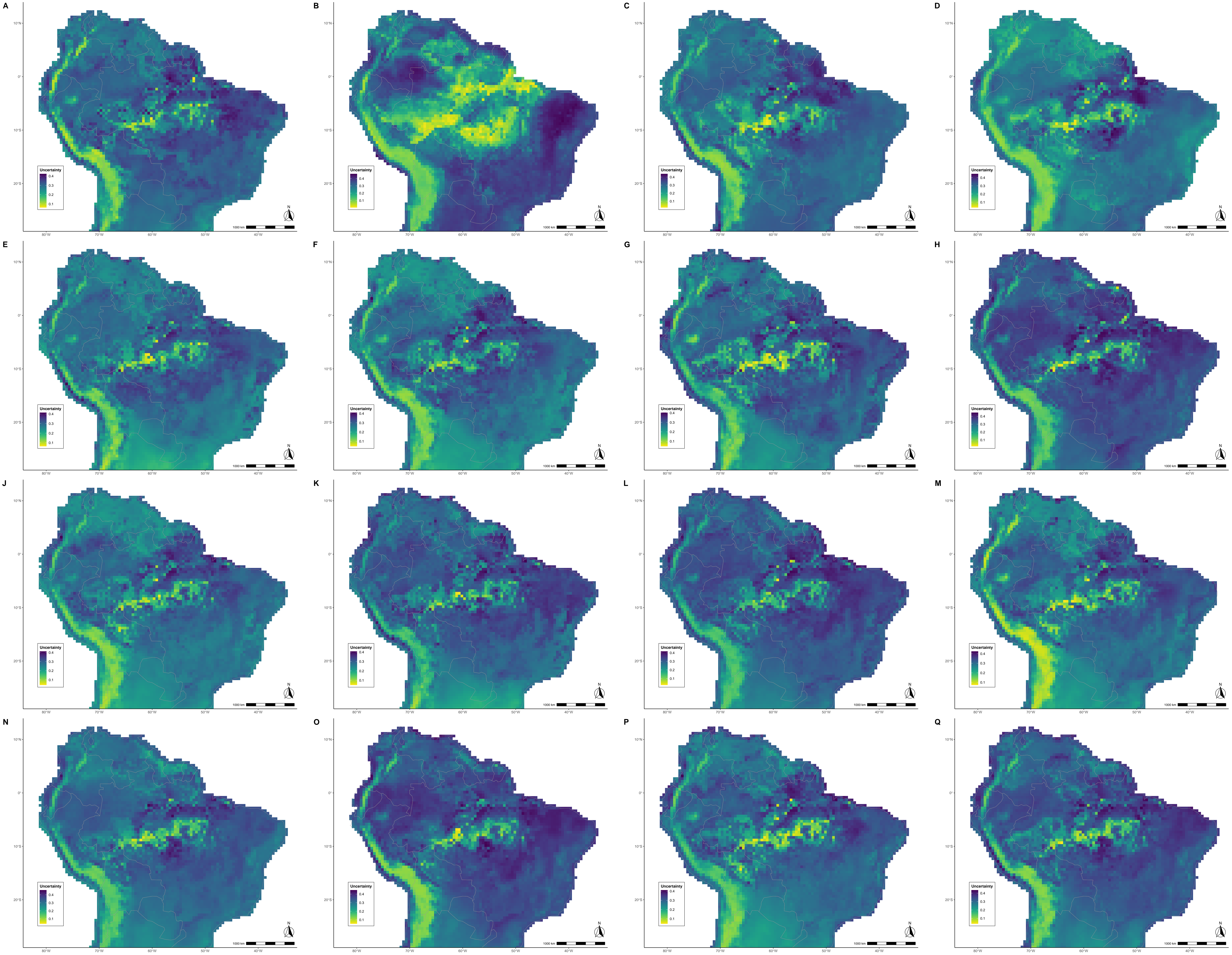

Supplement: Supplementary file 8 — Figure S3: Maps of areas gained and lost in Last Glacial Maximum (LGM) and in every 10 years in near‐term future (2021–2040) under 3–7.0 (moderate) and 5–8.5 (worse scenario) Shared Socioeconomic Pathways (SSPs) relative to the current potential distribution of D. micrantha , D. odorata , D. polyphylla, D. punctata , and D. rosea to demonstrate the pattern of migration of Dipteryx species. [file ECE3-15-e72105-s002.pdf]

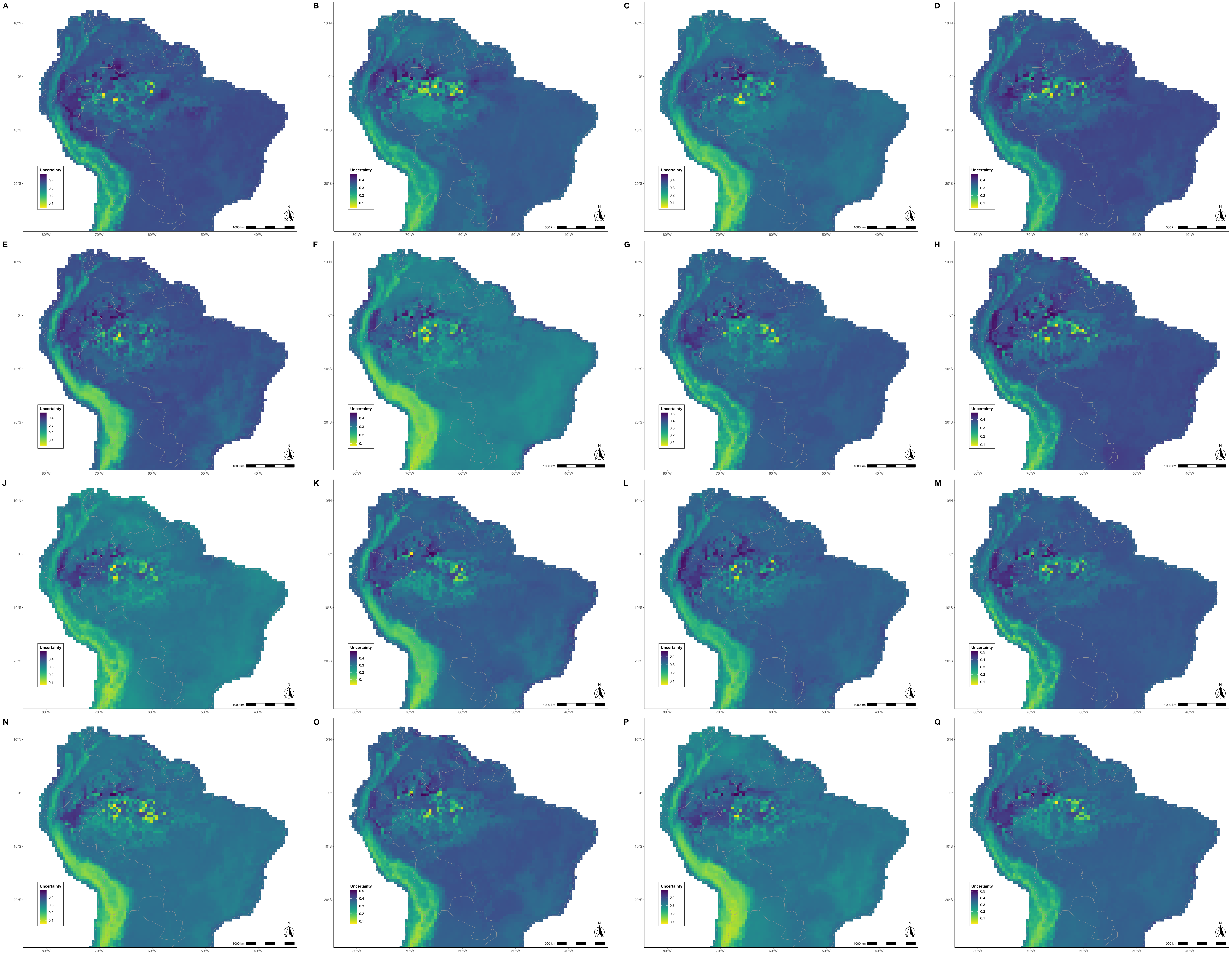

Supplement: Supplementary file 9 — Figure S4: Maps of uncertainty between the General Circulation Models (GCMs) MPI‐ESM‐P, MIROC‐ESM, and NCAR CCSM4 of Last Glacial Maximum (LGM) and between EC‐Earth3, MPI‐ESM1‐2‐LR, and IPSL‐CM6A‐LR for the near‐term future (2021–2040) assessed in every 10 years under 3–7.0 (moderate) and 5–8.5 (worse scenario) Shared Socioeconomic Pathways (SSPs) relative to the D. alata , D. charapilla, D. ferrea , D. lacunifera, and D. magnifica . [file ECE3-15-e72105-s003.pdf]

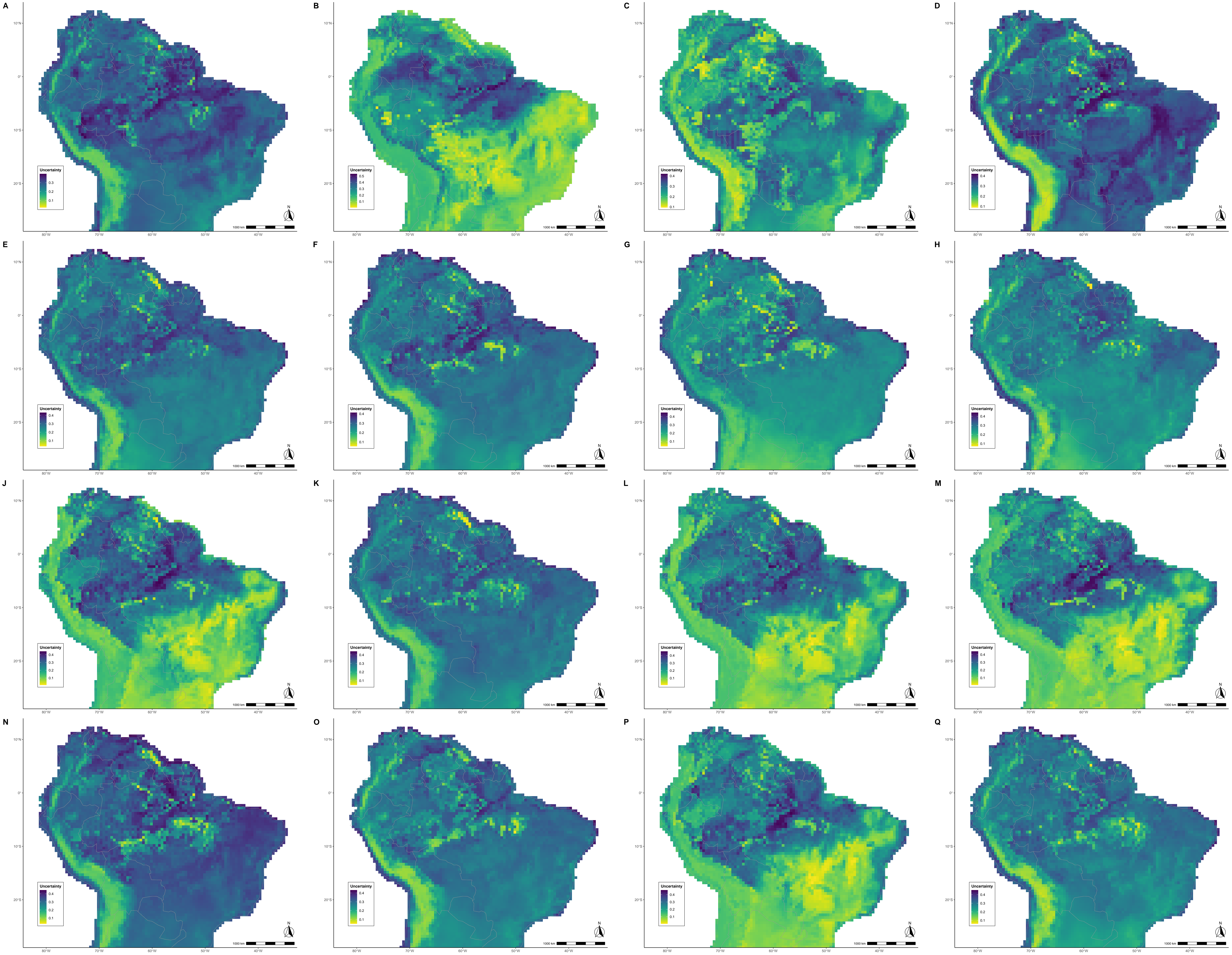

Supplement: Supplementary file 10 — Figure S5: Maps of uncertainty between the General Circulation Models (GCMs) MPI‐ESM‐P, MIROC‐ESM, and NCAR CCSM4 of Last Glacial Maximum (LGM) and between EC‐Earth3, MPI‐ESM1‐2‐LR, and IPSL‐CM6A‐LR for the near‐term future (2021–2040) assessed in every 10 years under 3–7.0 (moderate) and 5–8.5 (worse scenario) Shared Socioeconomic Pathways (SSPs) relative to the D. micrantha , D. odorata , D. polyphylla, D. punctata , and D. rosea . [file ECE3-15-e72105-s024.pdf]

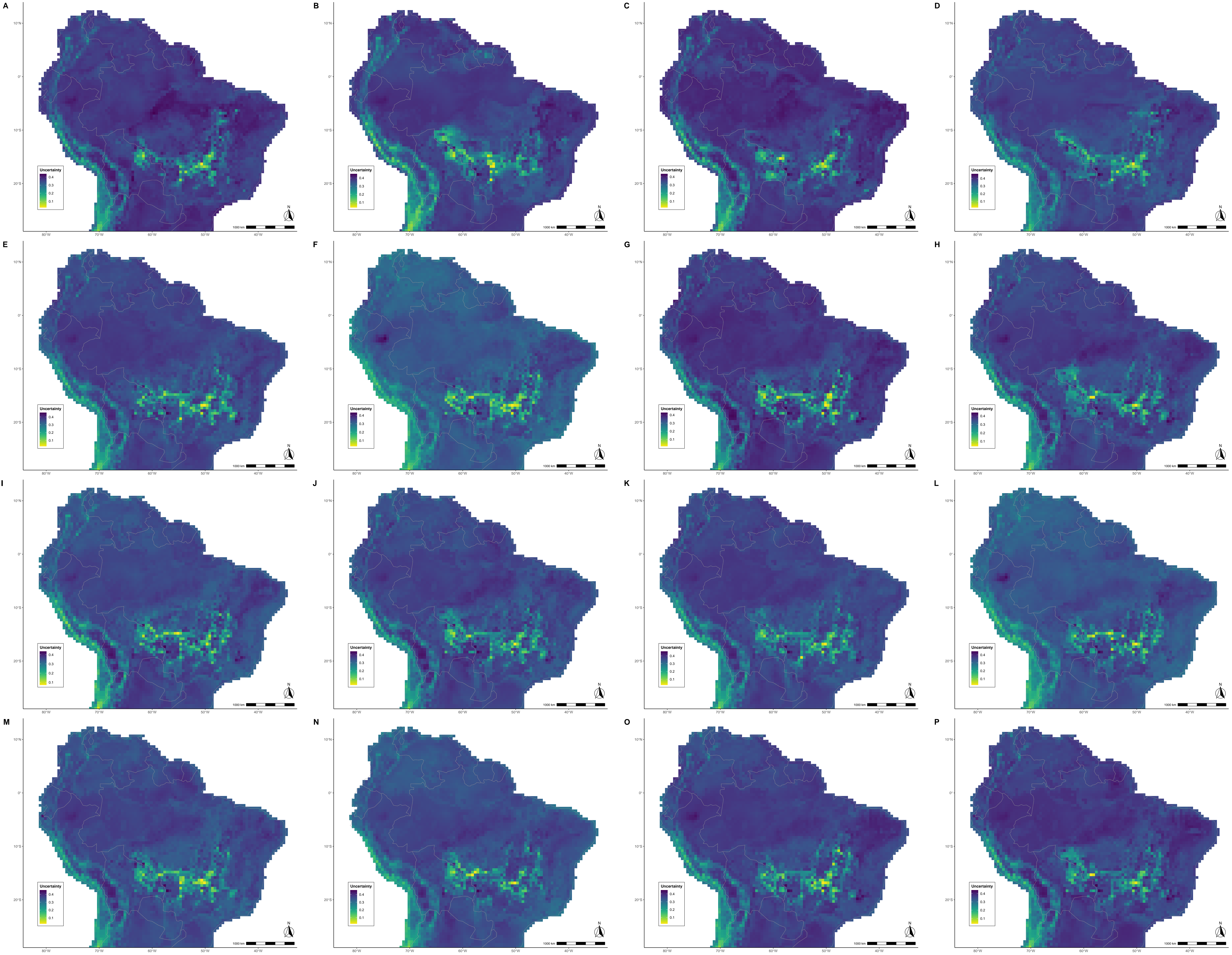

Supplement: Supplementary file 11 — Figure S6: Maps of uncertainty between the algorithm presence‐only (BIOCLIM and Domain), presence–pseudoabsence (SVM), and presence–absence (generalized linear model [GLM] and Random Forest) relative to D. alata . (A) Present days. (B–D) Last Glacial Maximum (LGM) General Circulation Models (GCMs): MIROC‐ESM (B), MPI‐ESM‐P (C), and NCAR CCSM4 (D). (E–P) Near‐term future GCMs under 3–7.0 (moderate) and 5–8.5 (worse scenario) Shared Socioeconomic Pathways (SSPs): EC‐Earth3, IPSL‐CM6A‐LR, and MPI‐ESM1‐2‐LR. [file ECE3-15-e72105-s017.pdf]

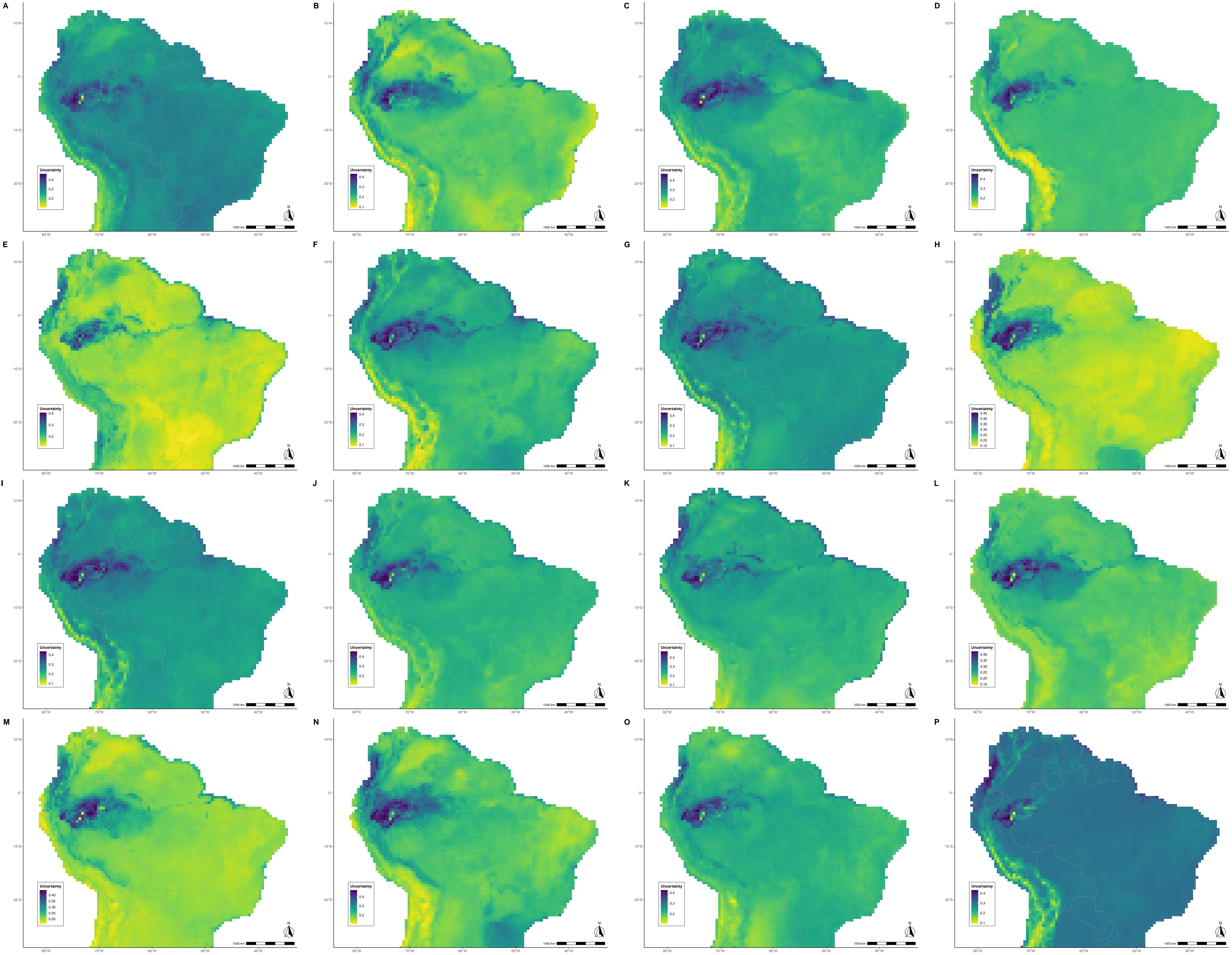

Supplement: Supplementary file 12 — Figure S7: Maps of uncertainty between the algorithm presence‐only (BIOCLIM and Domain), presence–pseudoabsence (SVM), and presence–absence (generalized linear model [GLM] and Random Forest) relative to D. charapilla. (A) Present days. (B–D) Last Glacial Maximum (LGM) General Circulation Models (GCMs): MIROC‐ESM (B), MPI‐ESM‐P (C), and NCAR CCSM4 (D). (E–P) Near‐term future GCMs under 3–7.0 (moderate) and 5–8.5 (worse scenario) Shared Socioeconomic Pathways (SSPs): EC‐Earth3, IPSL‐CM6A‐LR, and MPI‐ESM1‐2‐LR. [file ECE3-15-e72105-s010.pdf]

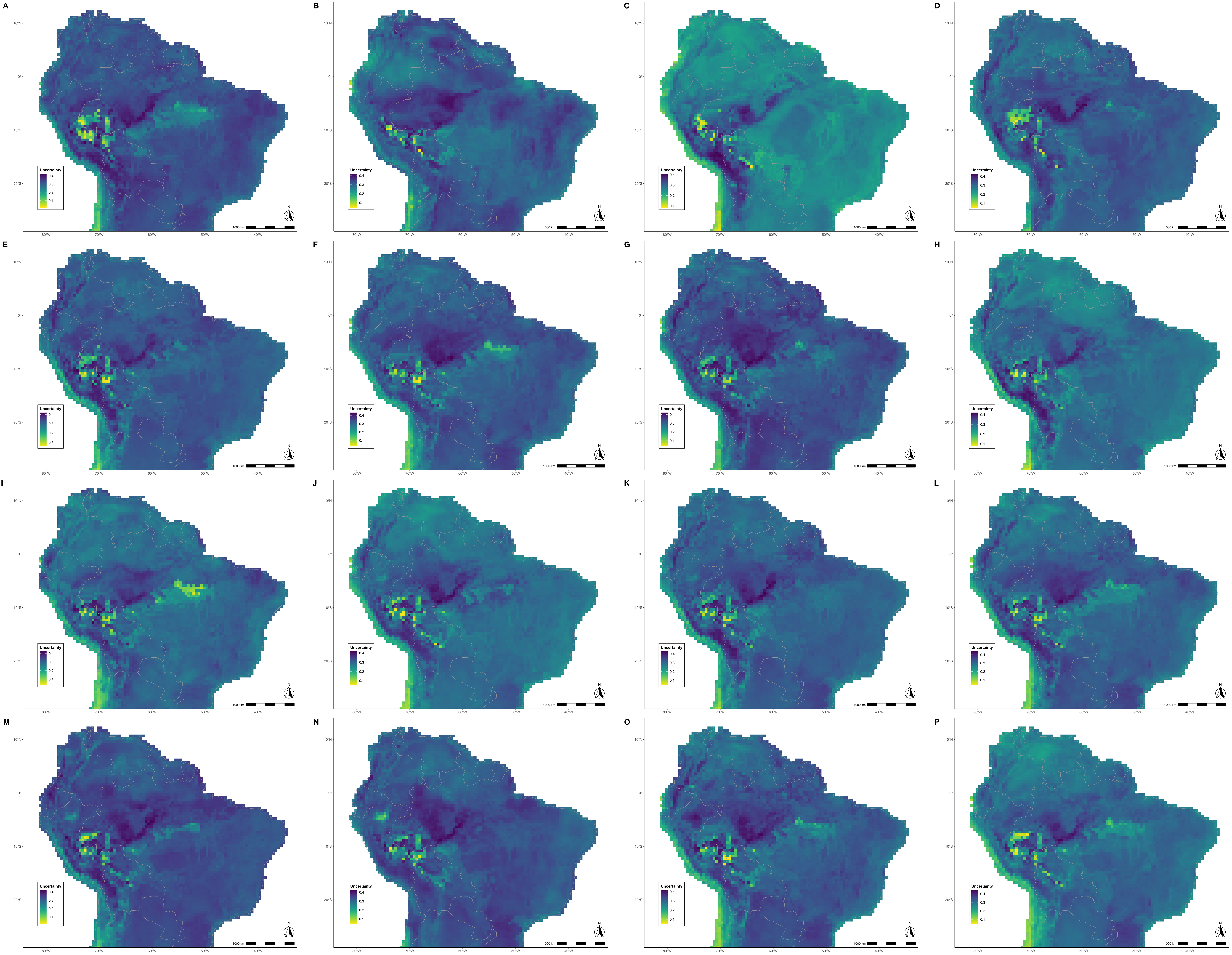

Supplement: Supplementary file 13 — Figure S8: Maps of uncertainty between the algorithm presence‐only (BIOCLIM and Domain), presence–pseudoabsence (SVM), and presence–absence (generalized linear model [GLM] and Random Forest) relative to D. ferrea . (A) Present days. (B–D) Last Glacial Maximum (LGM) General Circulation Models (GCMs): MIROC‐ESM (B), MPI‐ESM‐P (C), and NCAR CCSM4 (D). (E–P) Near‐term future GCMs under 3–7.0 (moderate) and 5–8.5 (worse scenario) Shared Socioeconomic Pathways (SSPs): EC‐Earth3, IPSL‐CM6A‐LR, and MPI‐ESM1‐2‐LR. [file ECE3-15-e72105-s011.pdf]

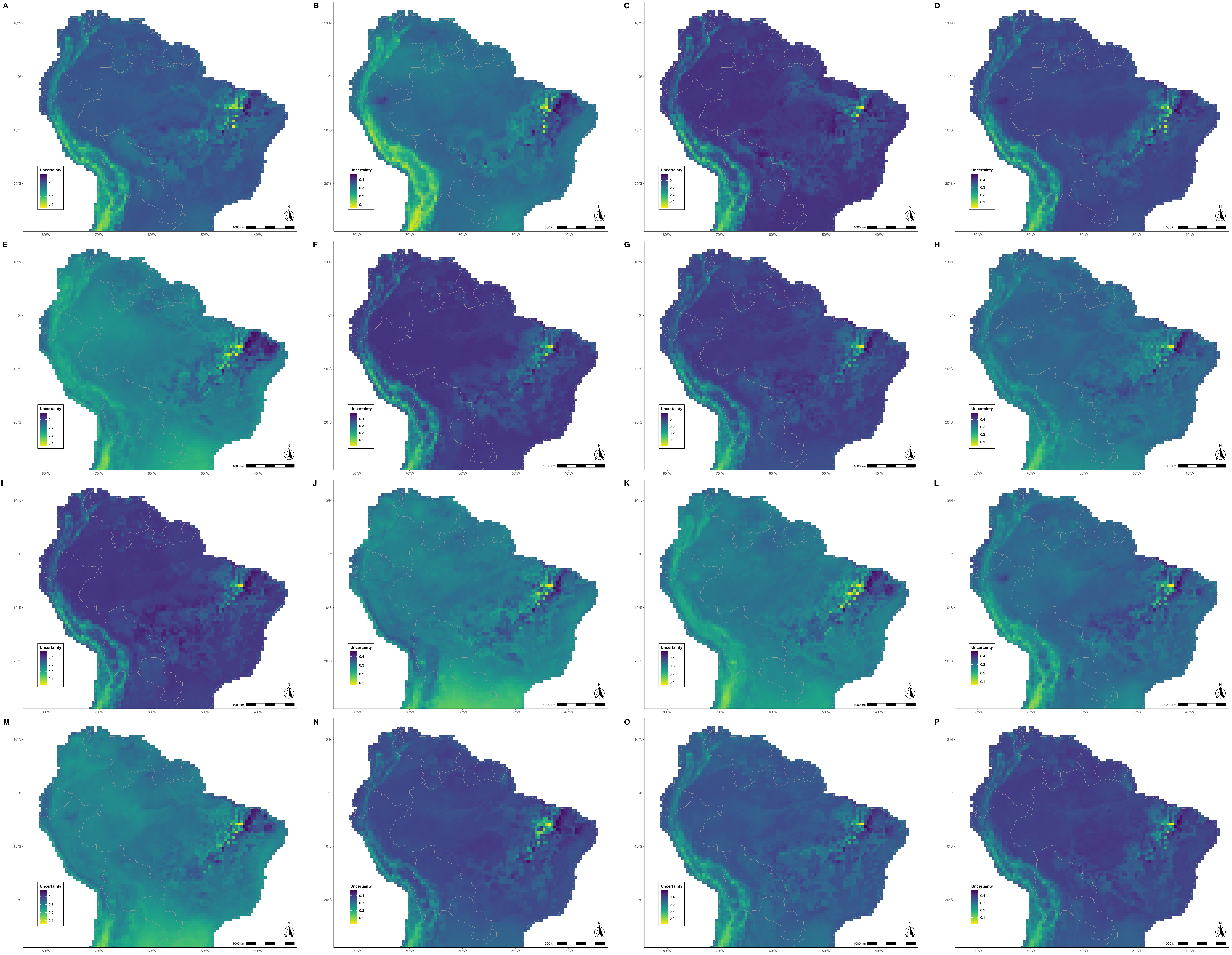

Supplement: Supplementary file 14 — Figure S9: Maps of uncertainty between the algorithm presence‐only (BIOCLIM and Domain), presence–pseudoabsence (SVM), and presence–absence (generalized linear model [GLM] and Random Forest) relative to D. lacunifera. (A) Present days. (B–D) Last Glacial Maximum (LGM) General Circulation Models (GCMs): MIROC‐ESM (B), MPI‐ESM‐P (C), and NCAR CCSM4 (D). (E–P) Near‐term future GCMs under 3–7.0 (moderate) and 5–8.5 (worse scenario) Shared Socioeconomic Pathways (SSPs): EC‐Earth3, IPSL‐CM6A‐LR, and MPI‐ESM1‐2‐LR. [file ECE3-15-e72105-s018.pdf]

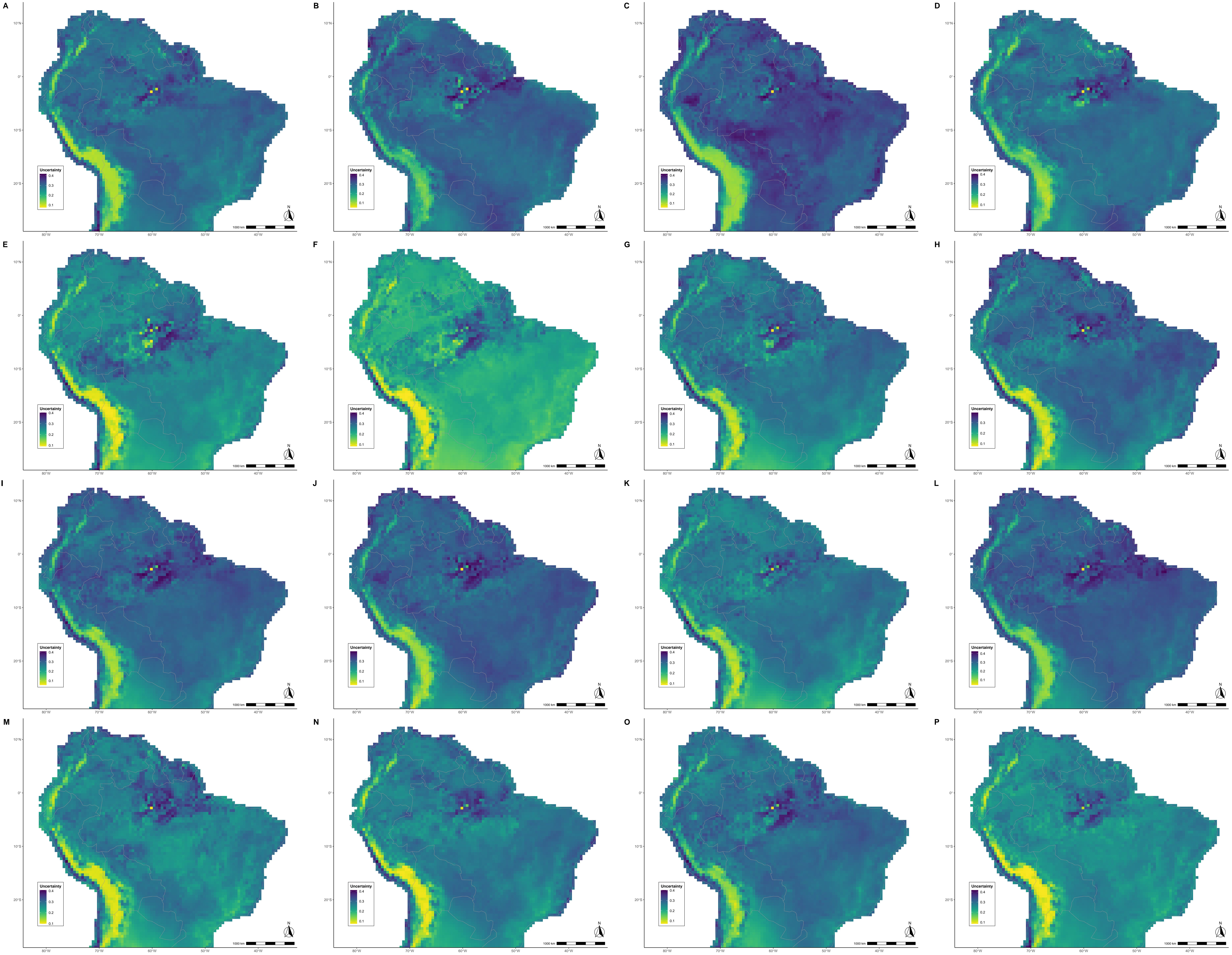

Supplement: Supplementary file 15 — Figure S10: Maps of uncertainty between the algorithm presence‐only (BIOCLIM and Domain), presence–pseudoabsence (SVM), and presence–absence (generalized linear model [GLM] and Random Forest) relative to D. magnifica . (A) Present days. (B–D) Last Glacial Maximum (LGM) General Circulation Models (GCMs): MIROC‐ESM (B), MPI‐ESM‐P (C), and NCAR CCSM4 (D). (E–P) Near‐term future GCMs under 3–7.0 (moderate) and 5–8.5 (worse scenario) Shared Socioeconomic Pathways (SSPs): EC‐Earth3, IPSL‐CM6A‐LR, and MPI‐ESM1‐2‐LR. [file ECE3-15-e72105-s004.pdf]

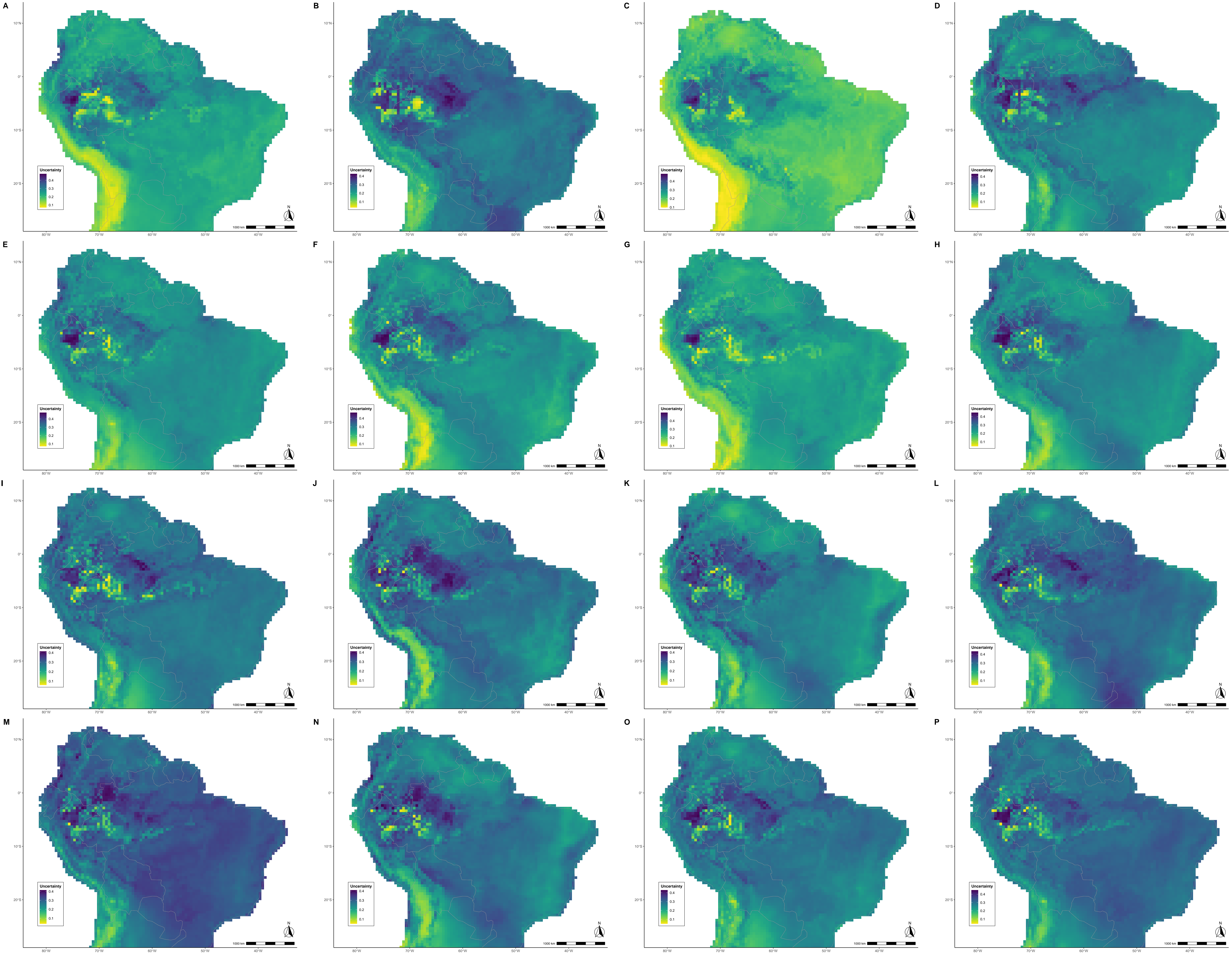

Supplement: Supplementary file 16 — Figure S11: Maps of uncertainty between the algorithm presence‐only (BIOCLIM and Domain), presence–pseudoabsence (SVM), and presence–absence (generalized linear model [GLM] and Random Forest) relative to D. micrantha . (A) Present days. (B–D) Last Glacial Maximum (LGM) General Circulation Models (GCMs): MIROC‐ESM (B), MPI‐ESM‐P (C), and NCAR CCSM4 (D). (E–P) Near‐term future GCMs under 3–7.0 (moderate) and 5–8.5 (worse scenario) Shared Socioeconomic Pathways (SSPs): EC‐Earth3, IPSL‐CM6A‐LR, and MPI‐ESM1‐2‐LR. [file ECE3-15-e72105-s005.pdf]

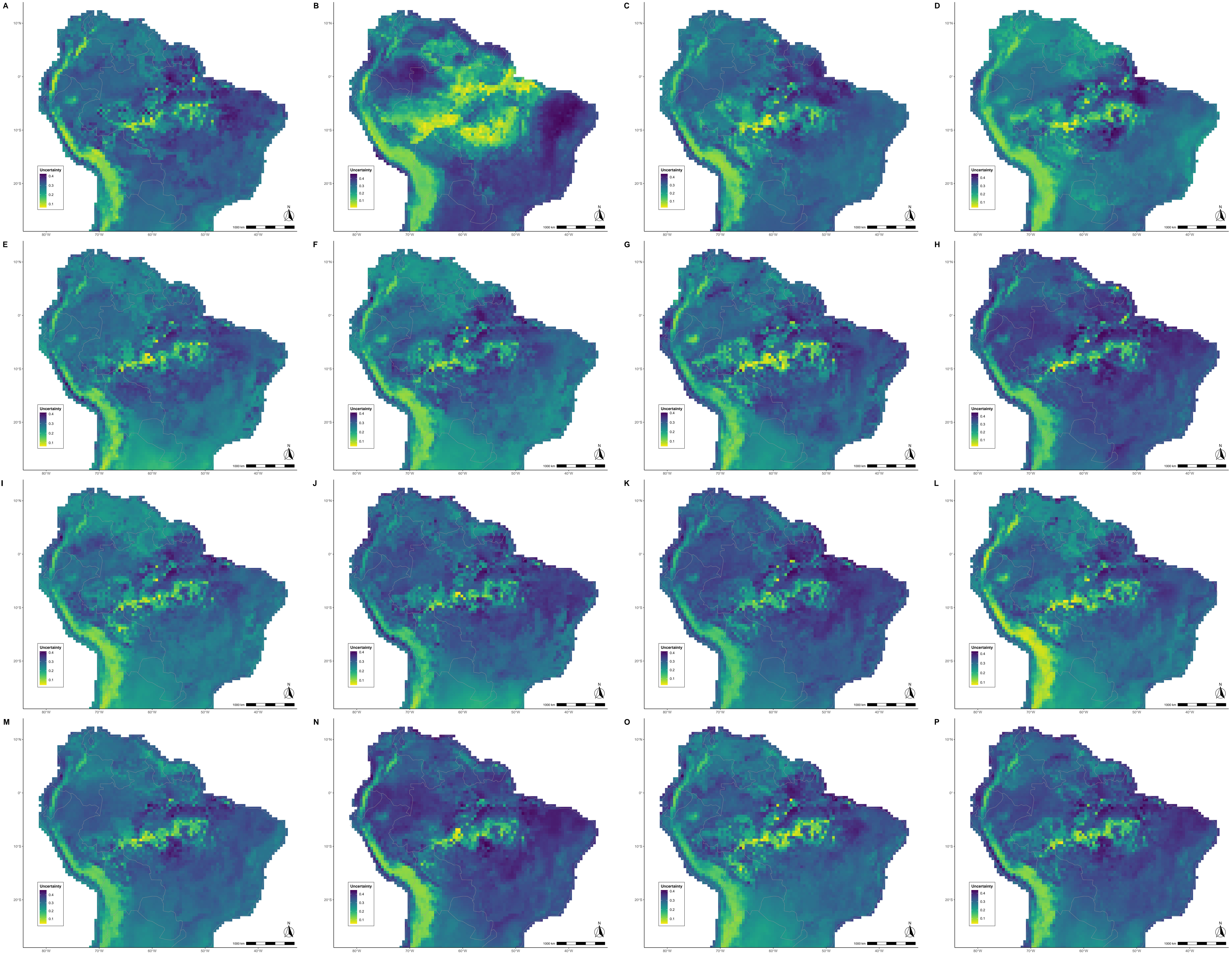

Supplement: Supplementary file 17 — Figure S12: Maps of uncertainty between the algorithm presence‐only (BIOCLIM and Domain), presence–pseudoabsence (SVM), and presence–absence (generalized linear model [GLM] and Random Forest) relative to D. odorata . (A) Present days. (B–D) Last Glacial Maximum (LGM) General Circulation Models (GCMs): MIROC‐ESM (B), MPI‐ESM‐P (C), and NCAR CCSM4 (D). (E–P) Near‐term future GCMs under 3–7.0 (moderate) and 5–8.5 (worse scenario) Shared Socioeconomic Pathways (SSPs): EC‐Earth3, IPSL‐CM6A‐LR, and MPI‐ESM1‐2‐LR. [file ECE3-15-e72105-s012.pdf]

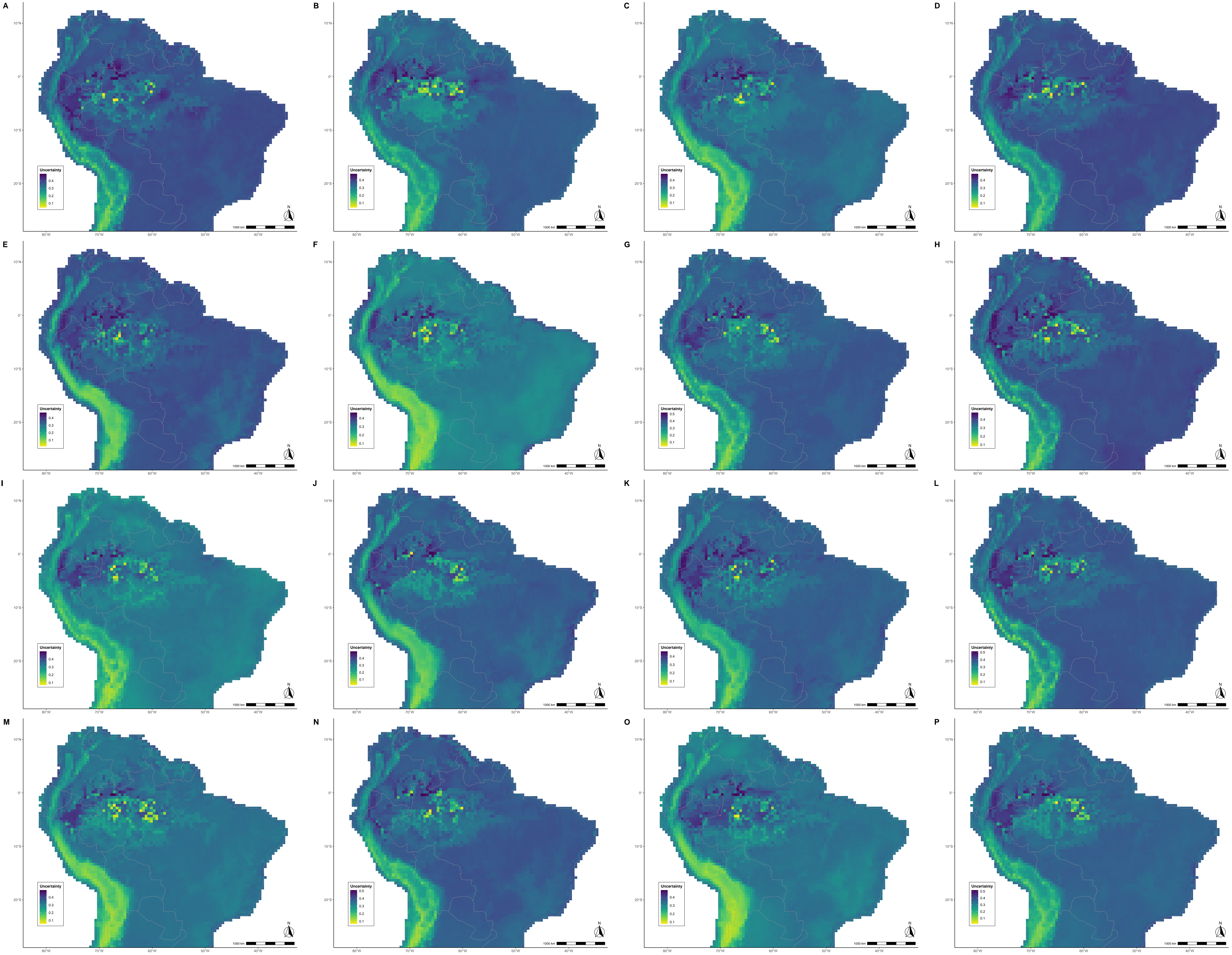

Supplement: Supplementary file 18 — Figure S13: Maps of uncertainty between the algorithm presence‐only (BIOCLIM and Domain), presence–pseudoabsence (SVM), and presence–absence (generalized linear model [GLM] and Random Forest) relative to D. polyphylla. (A) Present days. (B–D) Last Glacial Maximum (LGM) General Circulation Models (GCMs): MIROC‐ESM (B), MPI‐ESM‐P (C), and NCAR CCSM4 (D). (E–P) Near‐term future GCMs under 3–7.0 (moderate) and 5–8.5 (worse scenario) Shared Socioeconomic Pathways (SSPs): EC‐Earth3, IPSL‐CM6A‐LR, and MPI‐ESM1‐2‐LR. [file ECE3-15-e72105-s021.pdf]

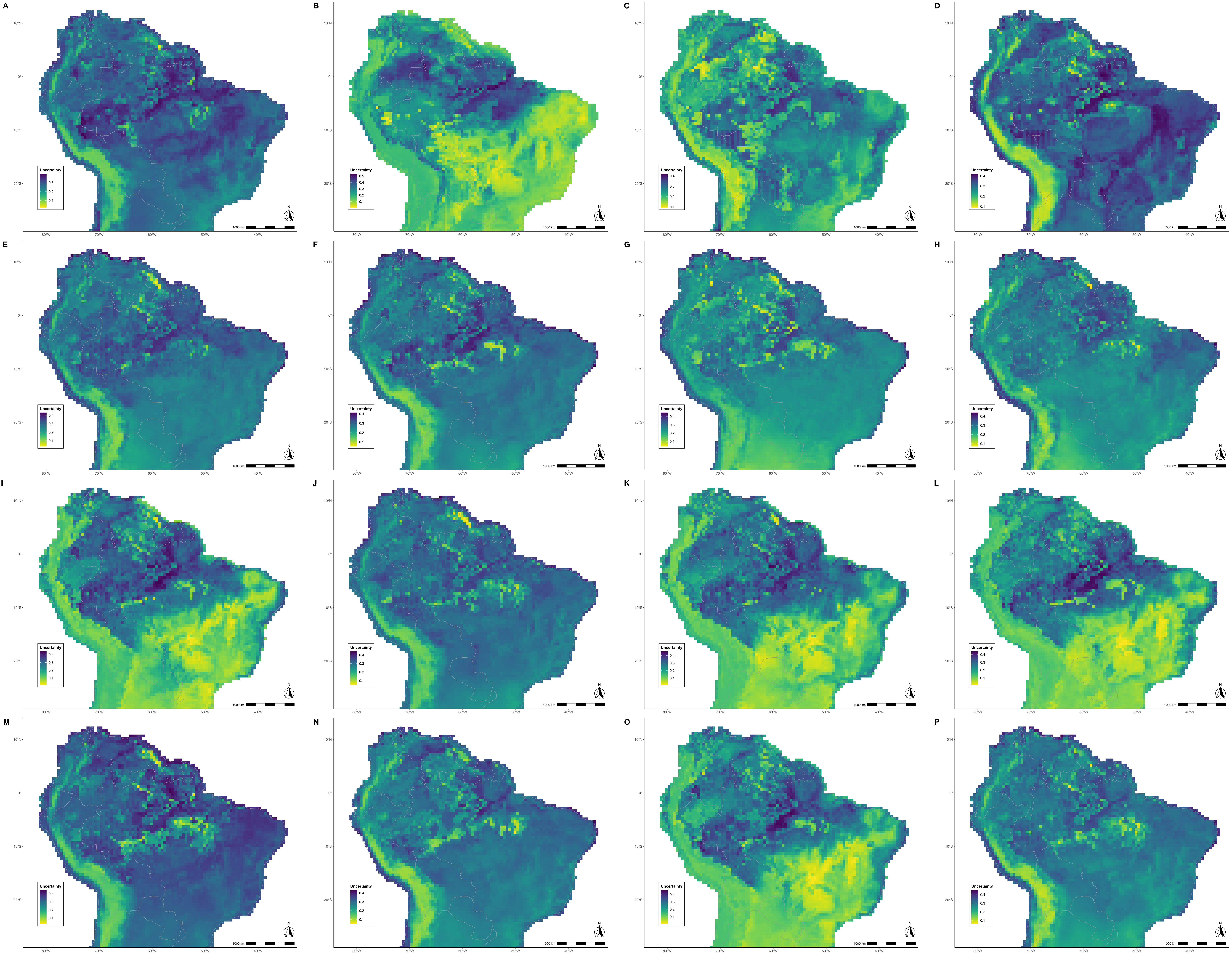

Supplement: Supplementary file 19 — Figure S14: Maps of uncertainty between the algorithm presence‐only (BIOCLIM and Domain), presence–pseudoabsence (SVM), and presence–absence (generalized linear model [GLM] and Random Forest) relative to D. punctata . (A) Present days. (B–D) Last Glacial Maximum (LGM) General Circulation Models (GCMs): MIROC‐ESM (B), MPI‐ESM‐P (C), and NCAR CCSM4 (D). (E–P) Near‐term future GCMs under 3–7.0 (moderate) and 5–8.5 (worse scenario) Shared Socioeconomic Pathways (SSPs): EC‐Earth3, IPSL‐CM6A‐LR, and MPI‐ESM1‐2‐LR. [file ECE3-15-e72105-s022.pdf]

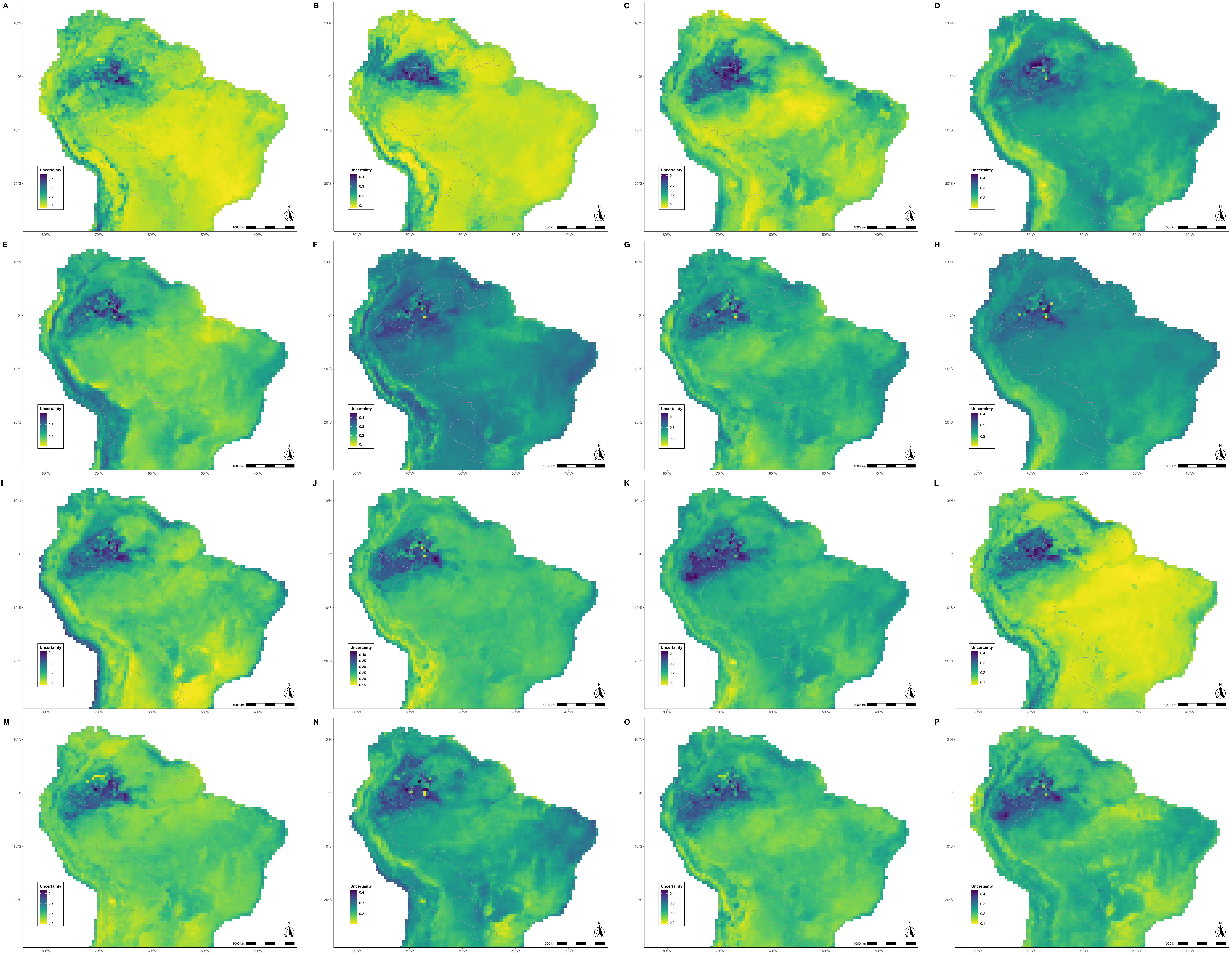

Supplement: Supplementary file 20 — Figure S15: Maps of uncertainty between the algorithm presence‐only (BIOCLIM and Domain), presence–pseudoabsence (SVM), and presence–absence (generalized linear model [GLM] and Random Forest) relative to D. rosea . (A) Present days. (B–D) Last Glacial Maximum (LGM) General Circulation Models (GCMs): MIROC‐ESM (B), MPI‐ESM‐P (C), and NCAR CCSM4 (D). (E–P) Near‐term future GCMs under 3–7.0 (moderate) and 5–8.5 (worse scenario) Shared Socioeconomic Pathways (SSPs): EC‐Earth3, IPSL‐CM6A‐LR, and MPI‐ESM1‐2‐LR. [file ECE3-15-e72105-s013.pdf]
